# Allocating organs through algorithms and equitable access to transplantation—a European human rights law approach

**DOI:** 10.1093/jlb/lsad004

**Published:** 2023-03-31

**Authors:** Audrey Lebret

**Affiliations:** Centre for advanced studies in Biomedical Innovation Law (CeBIL), Copenhagen University, Copenhagen, Denmark; Paris Human Rights Center (CRDH), Université Paris Panthéon-Assas, Paris, France

**Keywords:** algorithm, artificial intelligence, Europe, human rights, non-discrimination, organ transplantation

## Abstract

Digitization in transplantation is not a new phenomenon. Algorithms are being used, for example, to allocate organs based on medical compatibility and priority criteria. However, digitization is accelerating as computer scientists and physicians increasingly develop and use machine learning (ML) models to obtain better predictions on the chances of a successful transplant. The objective of the article is to shed light on the potential threats to equitable access to organs allocated through algorithms, whether these are the consequence of political choices made upstream of digitization or of the algorithmic design, or are produced by self-learning algorithms. The article shows that achieving equitable access requires an overall vision of the algorithmic development process and that European legal norms only partially contribute to preventing harm and addressing equality in access to organs.

## I. INTRODUCTION

In 2020, almost 60,000 patients were active on waiting lists for organ transplantation in the European Union (EU).^1^ Most organs are transplanted from deceased donors, but several European countries have developed living kidney and (part of) liver donation between siblings or persons with strong emotional ties. While so-called ‘altruistic’ donations between unrelated living donors and recipients are rarer, cross-donations programs between several donor/recipient pairs are contributing to reduce the gap, although far insufficiently, between need and supply of organs. Organ procurement,^2^ including the modalities of consent or authorization, and organ allocation are a matter of state law, but collaborative networks in Central Europe (Eurotransplant), Northern Europe (Scandiatransplant), and Southern Europe (South-Europe Alliance for Transplantation) enable transplantation centers to exchange organs between their respective Member States.^3^ These networks cannot solve the shortage on their own, but they do prevent the waste of organs. The EU has facilitated exchanges between EU Member States through the adoption of a directive in 2010, harmonizing the norms of quality and security of cross-border organ exchanges.^4^ For the purposes of transparency and safety, the directive imposes the collection of a minimum data set for each transplant.^5^ The directive imposes the same standards of protection when EU Member States transfer or receive organs from third countries (including through the above-mentioned networks).^6^

In order to manage this scarce resource at the national or international level, countries and above-mentioned networks use algorithms that help to find in a short time the best matches donor-recipient relying on criteria of medical compatibility and priority. Moreover, computer scientists and physicians are increasingly using machine-learning (ML) models for clinical transplantation, whether these models are supervised (when outcomes are defined) or unsupervised (when outcomes are not defined, the model will identify similar patterns in the dataset itself).^7^ Researchers develop and use ML to predict the likelihood to need a transplant within a given time; patient’s survival on the waiting list; graft survival or rejection after transplantation, including by learning from biopsy pictures, etc. Deep learning (DL) algorithms enable researchers to rely on sophisticated models of interactions between the characteristics of the organ and the recipients to provide a more suitable solution for patients waiting for transplants.^8^ The use of such trained algorithms could enable medical doctors to avoid ruling out a potential kidney transplant because of usual risks when the algorithm suggests that, in a patient’s particular situation, the transplant is likely to be successful. To carry out their studies, computer scientists use different types of algorithms, sometimes concomitantly, including artificial neural networks or random forests that are better at capturing the synergy between variables. The development of algorithms and in particular ML in clinical transplantation is promising for patients on the waiting list, increasing their chances to get a transplant, and avoiding the loss of donated organs.

Nonetheless, despite their incommensurate benefits, algorithms often lack of transparency and may produce unfair outputs that are difficult to detect. Therefore, the increasing development and use of algorithms in transplantation calls for scrutiny of how algorithms are built and trained in order to achieve their goals and ensure equitable access to transplants.

Non-discrimination and equitable access to health resources are guaranteed by several European legal instruments and programs, at the level of both the Council of Europe and the EU. While the Council of Europe is the main international organization on Human Rights in Europe and composed of 46 States, the EU is a supranational political and economic union of 27 States.^9^ Although these two organizations have different foundations, they interact in multiple ways. In particular, there is a presumption of equivalent human rights protection.^10^ In November 2019, the committee on Bioethics of the Council of Europe adopted a strategic action plan on Human Rights and Technologies in biomedicine to promote, in particular, equity in healthcare.^11^ The action plan encourages Member States to ‘ensure that innovative treatments and technologies are available “in an equitable and timely manner”’ and to combat health disparities.^12^

With regard to the use of digital systems, studies have demonstrated the risks that the development of algorithms (especially ML or DL) could pose to fundamental rights, including biases leading to a breach of equality between patients.^13^ These concerns had led to the adoption of several guidelines and have been partly included in regulatory projects, especially the recent proposal for a European Regulation on Artificial Intelligence (Proposal of AI Act).^14^ However, it is important to stress that in most cases, algorithms extend risks rather than create them. Indeed, the risks exclusively related to the learning process when using self-learning algorithms are only a fraction of the threats to equitable access to transplantation. For this reason, the present article proposes to analyze their use and development in the field of organ transplantation in their socio-political context rather than focusing on the sole technical aspects.

The objective of the article is to shed light on the potential threats to equitable access to organs allocated through algorithms, whether these are the consequence of political choices made upstream of digitization or of the algorithmic design, or are produced by self-learning algorithms. In that respect, the primarily legal article will include broader issues of distributive justice or bioethics related to transplantation that pre-exist digital technology, insofar as, when transposed to algorithms, they may affect equitable access to organs such as framed by European human rights law. Besides, the article does not only focus on ML algorithms but also includes organ allocation algorithms that have been used for a longer time. Although inspired by research and practices in the United States, the analysis is based on European law. It will show that reaching an equitable access requires to have an overall vision of the algorithmic development process and that European legal norms only contribute partially to prevent harm and to address equality in access to organs.

Section II aims to provide a framework for analysis by providing elements of definition of ‘equitable access to organs’ in Europe while outlining the related state obligations. In order to do so, the section proceeds to a cross-reading of relevant European legal sources enriched by a health and human rights approach. On this basis, Section III explores how threats to equitable access may happen in the algorithmic context, and discusses, where appropriate, the gap between current legal standards and algorithmic reality. Such threats arise from the data itself or the ways to pre-process it or from the algorithmic development and deployment. At this point, subsections develop on the choices and/or constraints weighing on computer scientists that need to incorporate certain criteria to define what makes a good transplant candidate. Following this examination of the algorithmic architecture, the issue of correlations made by ML algorithms is then considered. This investigation results in conclusive remarks in Section IV on the interests of this human rights approach of the algorithmic allocation of organs and on the remaining challenges to achieve equitable access in Europe. It then suggests to delineate the need for upstream structural reforms and algorithmic due diligence. Some key state obligations are identified.

## II. A REALISTIC EUROPEAN LEGAL FRAMING OF EQUITABLE ACCESS TO ORGANS

Allocation of health resources and priority-setting is first a matter of state law. European human rights law provides certain realistic standards concerning the access to scarce biomedical resources (A) and protects the right to be free from discrimination, whatever the forms such discrimination may take. The interpretations are not, however, completely uniform between European bodies (B). In addition to the individual rights approach, a health and human rights approach allows to enrich the definition of equitable access in the particular context of organ transplantation (C).

### II.A. Access to Limited Biomedical Resources

In the field of biomedicine and human rights, most of the relevant legal instruments are from the Council of Europe. The Oviedo Convention on Human Rights and Biomedicine and its Additional Protocol on Transplantation of Organs and Tissues guarantee an equitable access to health care. According to the explanatory report of the Convention, ‘[i]n this context’, equitable ‘means first and foremost the absence of unjustified discrimination. Although not synonymous with absolute equality, equitable access implies effectively obtaining a satisfactory degree of care’.^15^ The Additional Protocol further endorses and requires allocation depending on waiting lists, and based on ‘transparent, objective and duly justified rules according to medical criteria’.^16^ The explanatory report of the Additional Protocol states that allocation should first be determined by ‘the fundamental principles of medical practice [that] apply in all countries’.^17^

In addition to these treaties specifically related to biomedicine and human rights, the European social Charter recognizes more broadly the right to protection of health.^18^ Besides, with the exception of Andorra, all Member States of the Council of Europe have ratified the UN International Covenant on Economic, social, and cultural rights, which guarantees the ‘right of everyone to the enjoyment of the highest attainable standard of physical and mental health’.^19^ The UN Committee on Economic, social, and Cultural rights (CESCR) provided a comprehensive general comment in which it establishes accessibility for all without discrimination as an inherent component of the right to health. It means that ‘health facilities, goods and services must be accessible to all, especially the most vulnerable or marginalized sections of the population, in law and in fact, without discrimination on any of the prohibited grounds’.^20^ They also need to be ‘acceptable’, ie ‘respectful of medical ethics and culturally appropriate, ie respectful of the culture of individuals, minorities, peoples and communities, sensitive to gender and life-cycle requirements’. Together with non-discrimination and acceptability, physical accessibility, affordability, and availability of resources are ‘essential elements’ of the right to health^21^ that should be assessed in order to achieve equitable access to health goods and services.

With regard to state obligations in relation to health, it is important to note that these instruments also acknowledge the lack of resources and admit that States have some margin in the allocation of resources. This is in line with the case law of the European Court of Human Rights (ECtHR) on health issues. The ECtHR, located in Strasbourg, controls the respect of the European Convention on human rights and fundamental freedoms (ECHR) by the Member States of the Council of Europe. Although the ECHR does not include a right to health as such, the ECtHR has protected health as part as the quality of life under article 8 of the Convention. In Pentiacova and others v. Moldova, the Court found inadmissible the applicants’ claim that the insufficient state funding of haemodialysis and the failure to cover their travel costs amounted to a violation of Article 8. The Court considered that the state margin of appreciation to assess priorities was higher when it came to allocating scarce resources.^22^ This jurisprudence is consistent with the progressive realization of the right to health,^23^ due to difference in resources between countries. Organ transplantation, including from living donors, requires extensive medical expertise and investment in research, and European countries have not developed these programs in the same way and to the same extent. The differences are also related to various cultural and ethical perceptions on organ retrieval on a living or deceased person.^24^ Indeed, organs are not simply a limited health resource, they are a biological resource the availability of which requires prior authorization to access a donor's body. The first objective of the above-mentioned treaties of the Council of Europe is to protect the rights and dignity of potential living or deceased donors in the field of transplantation. Living donations for instance are often subsidiary to deceased donations and require approval of an independent body.^25^ Although a dead person has no legal personality, some of her rights extend after death and European human rights law protects the human body after death.^26^ Organs are therefore not ‘available’, which reinforces the states’ margin of appreciation in managing this resource.

Nevertheless, States must take reasonable steps^27^ to resolve the resource allocation dilemma. Progressive realization does not mean elusive obligations, and the CESCR made it clear that some obligations ‘such as the guarantee that the right will be exercised without discrimination of any kind (art. 2.2) and the obligation to take steps (art. 2.1) towards the full realization of article 12’ are of immediate effect.^28^ In its general comment, the CESCR listed among the core obligations ‘to ensure the right of access to health facilities, goods and services on a non-discriminatory basis, especially for vulnerable or marginalized groups’ but also ‘[t]o ensure equitable distribution of all health facilities, goods and services’.^29^

### II.B. The Absence of Discrimination

Apart from being an essential component of the right to health, non-discrimination is a freedom that European law protects either in conjunction with other specific rights, or as such. This section introduces the relevant European instruments, before comparing the prohibited grounds for discrimination, the forms, and potential justification of differential treatments based on protected characteristics. These elements will be relevant for the later analysis of the risks in the algorithmic context.

Although EU primary law addressed equality and non-discrimination in several treaties, the treaty of Lisbon, which entered into force in 2009, greatly contributed to the promotion of equality in the EU by giving a binding nature to the 2000 EU Charter of Fundamental Rights.^30^ The EU Charter guarantees equality before the law (article 20), non-discrimination (article 21), and equality between women and men (article 23). The Charter applies to EU institutions, office and agencies, and only to EU Member States when they implement EU law, such as the future European Regulation on AI (AI Act Proposal) concerning high-risks AI systems.^31^ Secondary law instruments give expression to the above-mentioned primary law provisions.^32^ Among those, several are directly and indirectly relevant to the issue of equitable access to organ transplants. Directive 79/7/EEC and Directive 2004/113/EC prohibit sex discrimination in, respectively, statutory social security schemes and access to goods and services, which applies in the field of health according to the Court of Justice of the EU (CJEU, located in Luxembourg).^33^ The Racial Equality Directive applies in relation to social protection including healthcare.^34^

At the Council of Europe level, Article 14 ECHR prohibits discrimination in conjunction with other convention rights, such the right to private life, which—as previously stated—has incorporated the protection of health. The adoption of Protocol 12 to the ECHR in 2000 integrated a general freedom not to be discriminated against within the Council of Europe system. The Protocol entered into force in 2005 and is applicable in 20 countries to that date. The European collaborative algorithms the next parts discuss are often used in non-contracting States of the Protocol.^35^ These countries remain bound by the ECHR, which guarantees the right to life and the right to private life, both requiring non-discrimination. The catalogue of—essentially—civil and political rights of the ECHR is completed by the European Social Charter, which contains the obligation to treat nationals of other contracting parties on an equal footing with own citizens concerning the right to social and medical assistance (article 13(4)) and a general prohibition of discrimination: article E.^36^

Article 14 ECHR and Article 1(1) Protocol 12 prohibit discrimination based on a non-limitative list of protected characteristics: ‘…any ground such as sex, race, colour, language, religion, political or other opinion, national or social origin, association with a national minority, property, birth or other status.’ The ECtHR’s jurisprudence interpreted ‘other status’ as including disability, medical conditions or genetic features,^37^ gender identity, sexual orientation, immigration status, and age to some extent.^38^ ‘To some extent’ because the Court’s scrutiny is not uniform for all protected grounds. Differential treatments based on age are not as strictly scrutinized as discrimination based on sex, sexual orientation, or race/ethnicity.^39^ The list of non-limitative protected characteristics in the European Social Charter is mostly similar to that of the ECHR but contains explicitly health as a protected ground. On the EU side, the EU Charter’s list of protected characteristics is non-limitative but broader than the ECHR ‘on any ground such as sex, race, colour, ethnic or social origin, genetic features, language, religion or belief, political or any other opinion, membership of a national minority, property, birth, disability, age or sexual orientation’.^40^

Concerning the forms of discrimination, the CJEU, the ECtHR, and the European Committee on social Rights, which monitors compliance with the European Social Charter in the Council of Europe,^41^ all recognize that discrimination may take multiple forms and all prohibit direct and indirect discrimination. *Direct discrimination* consists in a differential treatment based on a protected characteristic. *Indirect discrimination* occurs when apparently neutral provisions or measures have a prejudicial effect in practice against a specific protected group.^42^ Indirect discrimination and, at least in part, direct discrimination do not require the intent to discriminate in European law.^43^ These two forms of discrimination have the potential to cover developers’ own biases but also the use of biased data, even when developers did not intend to discriminate but designed a model producing a disadvantage to a protected group.

In addition, both European Courts recognized the notion of *discrimination by association*, to persons ‘although not themselves a member of the [protected] group concerned, nevertheless suffer less favourable treatment or a particular disadvantage on one of those grounds’^44^ because of a discriminatory treatment against a person they are usually closely connected too.^45^

Despite this common base, the European Courts’ jurisprudence diverge in some regards to the point that some authors talk about ‘invalidating jurisprudential diversity’.^46^ One significant difference is on ‘intersectional’ forms of discrimination. EU law recognizes the existence of multiple discrimination but not ‘intersectional’ discrimination.^47^ In contrast with multiple discrimination that can be additive and cumulative, intersectional discrimination cannot be captured on one single protected characteristic but rather emerges from the interplay between different grounds.^48^ By centralizing these various forms of oppression, intersectionality theory offers a reading of non-discrimination law in light of ‘a new philosophy of *inequality*’.^49^ To this date, the European Courts did not formally endorse such broadening of the scope of non-discrimination law. However, while the CJEU has a mechanical interpretation,^50^ the ECtHR has a pragmatic and dynamic approach. Although the ECtHR does not directly consider intersectional discrimination, it analyses concomitant forms of discrimination or disparities in connection to a broad and casuistic concept of vulnerability.^51^ Beyond the advantages and weaknesses of intersectionality theory,^52^ the concept of intersectional discrimination has great potential for addressing the risks associated with ML algorithms, which rely on complex forms of interactions between medical and social characteristics of patients. The vulnerability approach does not provide clear standards to prevent discrimination in algorithmic systems.

The proof of non-discrimination is based on a test of comparability of situations, whether direct or indirect discrimination is at stake. If the situations are too different to be comparable, non-discrimination law is not applicable. The analysis of analogy between situations is factual and the interpreter has a broad margin.^53^ The way to formulate the respective situations and identify the reference circle for comparison determines their degree of similarity, which consequently affects the scrutiny of the legitimacy and necessity of the challenged treatment. Qualification thus carries political weight.^54^

When situations are comparable, ie identical or slightly identical (and there is a differential treatment, with the risk of direct discrimination) or very different (and there is a similar treatment, leading to a potential indirect discrimination), the ECtHR and the European Committee of Social Rights will analyze the justification of this treatment. This contrasts with EU law on direct discrimination, which usually excludes the justification in the context of direct discrimination.^55^ In this regard, an applicant claiming to be victim of direct discrimination on the basis of ethnicity would have better chances of success in Luxembourg (CJEU) then in Strasbourg (ECtHR). Nevertheless, EU law does not have a uniform approach and the directive related to equal treatment between men and women in the access to and supply of goods and services, allows for some justification also in case of direct discrimination,^56^ stating that the ‘directive shall not preclude differences in treatment, if the provision of the goods and services exclusively or primarily to members of one sex is justified […]’. Whether required by indirect discrimination only or both direct and indirect discrimination, such justification consists in the presence of a ‘legitimate aim’ and ‘proportionality between that aim and the means employed’.^57^

Where the CJEU, the ECtHR, or the European Committee of Social Rights have found that situations were different yet still comparable, they have identified positive state obligations to address these differences in their interpretation of non-discrimination.^58^ In the wording of the Court of Strasbourg, States need to treat differently persons or groups whose situations are ‘significantly different’ unless they can provide an objective and reasonable justification for their failure to do so.^59^ In the case of persons with disabilities, such measures to ensure substantive equality and combat discrimination constitute a state obligation of ‘reasonable accommodation’.^60^ Nevertheless, European approaches are not homogeneous either with regard to the basis and the extent of these positive obligations to address the particular circumstances of certain individuals or groups. The European Committee of Social Rights has a more assertive approach than the ECtHR. Previously associated with indirect discrimination, the failure to take positive action has gained autonomy in the Committee’s decisions and has become a form of discrimination in itself.^61^ Faced with a difference of situation in the exercise of social rights, the European Committee has imposed a ‘reinforced obligation of means’^62^ on States to make ‘measurable progress and to an extent consistent with the maximum use of available resources’.^63^ In contrast, the jurisprudence on non-discrimination reveals that the ECtHR is more hesitant to require a general obligation to take substantial positive measures to redress factual differences.^64^ The ECtHR specified that States had positive obligations when people or groups’ circumstances were ‘relevantly and significantly different’,^65^ providing that

relevance is measured in relation to what is at stake, whereas a certain threshold is required in order for the Court to find that the difference in circumstances is significant. For this threshold to be reached, a measure must produce a particularly prejudicial impact on certain persons as a result of a protected ground, attaching to their situation and in light of the ground of discrimination invoked.^66^

When States take specific measures favoring certain groups in order to correct and compensate a pre-existing disadvantage (ex: quotas), the CJEU insisted on the need of proportionate ‘special measures’, which means they cannot introduce an automatic and unconditional priority.^67^

To summarize, European law offers protection against multiple forms of discrimination on non-limitative grounds, but the extent of scrutiny varies depending on the protected ground. While it does not formally recognize ‘intersectional’ discrimination, the vulnerability approach of the ECtHR enables it to capture multiple and concomitant forms of discrimination, although not systematically. A risk of broadening the scope of non-discrimination would be to inflate the notion to the point where it loses its function of protecting groups particularly at risk. However, neither the EU nor the ECtHR’s approaches currently provide clear operating standards to address the risk of discrimination based on complex forms of interactions, which is one of the higher risks of ML. Uncertainties about the extent of positive obligations to redress inequalities within a non-discrimination framework may be offset by an additional health and human rights-based approach to organ allocation.

### II.C. A Health and Human Rights Approach to Organ Allocation

European law on biomedicine and human rights is pragmatic when it comes to delineating the scope of a right to access human organs for transplantation, being primarily understood as the absence of discrimination. Human rights law does not provide a uniform definition of equitable access, being rather concerned about the ‘fair deliberative process’ when it comes to setting priorities in health.^68^ Nonetheless, this framing of equitable access can be further refined by a ‘health and human rights approach’ to access to transplantation.^69^ Such a reading allows for the consideration of relevant socio-determinants of health where non-discrimination law relies on protected characteristics to be applicable and does not (at least yet in the European context) fully capture intersecting causes of vulnerability due to the Courts’ single-axis approach. Instead of the binary approach of human rights *subjects* (individual/vulnerable groups broadly defined), social determinants of health interestingly complete this framing by addressing social disparities more gradually: ‘to reduce the steepness of the social gradient in health, actions must be universal, but with a scale and intensity that is proportional to the level of disadvantage’.^70^ Socio-determinants of health include social and cultural factors, physical environment, income, access to education and health and social services, and abilities to be involved and participate in societal debates. Merging the respective approaches, the UN CESCR incorporated the social determinants of health in its interpretation of the right to health.^71^ In human transplantation, the path to equality in access to transplant cannot overlook the disparities in transplant knowledge and education, diagnosis, access to waiting lists, death rates before or while on the waiting lists depending on multiple—and interacting—factors such as geographical location, poverty, race, gender, or disability.^72^ Socio-demographic factors also play on the acceptance and functioning of transplant.^73^

Investigating socio-determinants of health can help to better understand geographic disparities in access to transplantation. At the transnational level, the Eurotransplant algorithm ensures a ‘genuine distribution across the participating countries’.^74^ Disparities also exist at the intrastate level. They may result from differences in the quantity and quality of transplantation facilities and services between rural and urban areas. In areas where there are few transplant centers, this may also be a barrier to living organ donation and thus to access to transplantation. When geographic distance from a transplant center primarily affects ethnic minorities, accessibility to transplantation for people belonging to these minorities is affected.^75^

Yet, little work has been done to identify and mitigate these disparities and improved research in health equity in transplantation is needed.^76^ Such studies provide the tools to assess the overall situation of patients and are thus necessary to any comprehensive study on equality in the algorithmic allocation of human organs for transplantation. They enable developers to make informed decisions on whether to include certain disparity factors in mathematical models of organ allocation by assigning them respective weights; or whether to exclude other factors from the models, leaving public authorities to address structural issues upstream and independently of the design of an allocation model. Weighing may be considered as positive action, i.e. a way to treat differently patients found to be in a different situation, meeting some of the above-mentioned requirements of non-discrimination law.

This overview of relevant European legal standards relevant for achieving equitable access to scarce biomedical resources, enriched by a health and human rights approach, provides a compass for the development of acceptable digital solutions.^77^ Due to the sensitive nature of organ transplantation, the shortage of organs, and the political aspects of non-discrimination law, States usually have a wide margin of appreciation in the allocation of resources. Therefore, achieving equitable access within a European human rights framework will require to assess the situation of respective patients with due regard to their individual rights, a balance of interests, objective justification of allocation, and proportionality. Based on the above, the following section discusses some of the threats to equitable access during the algorithmic development.

## III. THE THREATS TO EQUITABLE ACCESS TO TRANSPLANTATION IN ALGORITHMIC DEVELOPMENT

Threats to equitable access can arise at different steps of the overall algorithmic development. This section analyzes the interactions between practices during the algorithmic development and the legal requirements above-mentioned. It first considers potential risks arising from the data feeding self-learning algorithms (A), second, those related to the design choices and constraints during the development of the algorithm (whether ML or not) (B), and third, the threats coming from the learning process when ML is used (C). Certain practices, inactions or processes can undermine the principle of equity and even constitute direct or indirect discrimination in access to transplantation. The section also shows that these current legal standards are not always fully adequate to deal with these respective algorithmic risks.

### III.A. Data Input and the Breach of Equality between Patients in Predictive Models

The following sub-sections focus on the data input to ML algorithms and examine how potential threats may emerge from two successive steps preceding algorithm development: data collection (1) and data pre-processing (2), and briefly conclude on the capacity of proposed regulatory frameworks to address the data challenge (3).

#### III.A.1. Data collection: unrepresentative data and inequitable predictions

One of the main challenges for a ‘fair AI’ is to bring more transparency on the collection of data on which data scientists will build and train medical algorithms. ML algorithms learn from the collected data and the higher the quantity of data, the more accurate will be the predictions. Moreover, equitable algorithmic outputs require representative and inclusive data input. At least, two inter-related issues on data collection need to be addressed in this regard: the fact that certain data are missing on the one hand (a), and the importation and/or use of inadequate data on the other hand (b).

##### III.A.1.a. Lacking data on certain groups

ML algorithms are built on an initial data set that scientists will generally split in three categories: the training data set (the largest set), the cross-validation set (or development set), and the test data set. For the use and development of predictive algorithms in medicine, it is crucial to have inclusive data in order to allow ML systems to learn and provide the same quality of prediction for all patients.^78^ Therefore, inclusive data for clinical transplantation imply to incorporate such relevant data enabling predictive outputs of equivalent quality for different population groups. However, such data are sometimes lacking, or in a reduced quantity in comparison with the majority population. Lacking data can result from factual considerations and the complexity of having enough representing data due to a limited amount of certain minority populations in a given context. This risk is the ‘sample size disparity’.^79^ This disparity can also result from structural and/or social factors disadvantaging certain groups including indigenous peoples or women. To give a relevant example on the former in the European context, international agencies but also political institutions within the Sami society have criticized the absence of state-collected Sami population data, leading to a very limited knowledge of the Sami health situation.^80^ Such structural issues on health data also concern women. In her influential book *Invisible Women*, Caroline Criado Perez explains how women were historically excluded from medical research, resulting in data gaps.^81^ In particular, clinical trials data on pregnant women are very rare.^82^ Even when women were not excluded from research, their specificities were not always considered or deemed relevant. Although there are gender differences in mechanical workings of the heart, lung capacity, immune systems, and sex-based differences in physiology, these differences were broadly overlooked.^83^ According to the CESCR in its general comment on the right to health, ‘as women are underrepresented in scientific research, it is very common that scientific research and new technologies are gender biased and not sensitive to the particularities and needs of women’,^84^ that is why it recommends a gender-based approach in health policies, ie an approach that ‘recognizes that biological and socio-cultural factors play a significant role in influencing the health of men and women’.^85^

Besides, an imbalance of data in a specific procedure such as organ transplantation may result from various and intertwining factors of disparities in access to waiting lists and transplantation. Addressing the lack of data requires looking upstream for the above-mentioned determinants of health and investigate, inter alia, perceptions of transplantation between different groups of individuals. For instance, the literature has shown that the ‘typical recipient is a male’, while women are the typical living donors.^86^ Disparities may appear early in the process since studies have shown that women and girls access less often to waiting lists and preemptive transplantation then men.^87^ Medical factors alone do not explain these differences, and both patient motivation and physicians and parental attitude toward transplantation may contribute to the disparity.^88^ Social norms regarding caregiving responsibilities, stereotypes, economic disadvantage, or lack of education and health literacy strongly contribute to the many challenges women with chronic kidney disease face.^89^

##### III.A.1.b. Using inadequate data

The use of algorithms in a certain context will only be relevant if data they are fed with represent the targeted patients. Factors such as lifestyle and traditions, beliefs, and medicinal knowledge may affect peoples’ health, expression of pain, and likeliness to consult a doctor.^90^ Their absence of inclusion in the data may lead to ineffective health predictions for the populations concerned.^91^ This issue of the adequate input data may happen at various levels. At the country level for instance, when data emanate from a privileged socio-economic context leading predicting algorithms to disadvantage certain populations. Nicholson Price have shown the ‘contextual bias’ of algorithms trained in high-resource contexts, and not being adapted to more vulnerable groups.^92^ In such circumstances, the algorithms might provide a high quality of care in a given local context by relying on appropriate data in this particular context, but disparities in qualitative predictions may result from the process of translating the algorithms to another socio-economic context^93^ or the use of the algorithms at a broader scale. In order to develop effective ML models on organ transplantation and to address data gaps, researchers try to obtain larger amounts of data. Within the frameworks of data protection, one way for respective institutions to achieve this is to share their data in international registries. For example, Eurotransplant and Scandiatransplant have agreements with the ISHLT, the largest repository of heart transplant data.^94^ In the absence of such registries or agreements, models may be developed using external data although they are intended for use in other countries/regions. The availability and precision of the data from the United Network on Organ sharing (UNOS) on donor and recipient characteristics and their relationship may explain why it is regularly used outside the USA.^95^ The use of external data may raise two issues in relation to equitable access to transplantation: the adaptability of US data to other social and health contexts and where populations may differ significantly, and behind this, the availability, accuracy, and transparency of local data.^96^ These risks may be disproportionally affecting certain minorities, such as indigenous populations or immigrants. When Canadian teams feed and train AI models of transplantation on the basis of UNOS database, the health predictions for indigenous communities of Canada for example may be of a significantly lesser quality than for the majority of the population.

Lack of data and use of inadequate data is likely to lead to inequitable health predictions. If such predictions disadvantage certain groups, they may constitute an indirect discrimination on the ground(s) of gender, national minorities, or ‘other status’, including socio-economic status. It is arguable that within a country, the circumstances of certain groups are ‘relevantly and significantly’ different on health matters, requiring state authorities to take positive measures to make individuals’ right to be free from discrimination as such or in conjunction with their right to health (or right to life) effective.

#### III.A.2. Data pre-processing: the case of variable selection for organ transplantation

Another threat to equitable access to organ transplantation might come from the pre-processing of the data. Pre-processing includes data cleaning, data transformation and data reduction. Data cleaning is the handling of missing or noisy data. Data transformation encompasses various tasks including the selection of features in the data that will constitute the variables of the algorithms. Depending on the objective of the algorithms, data scientists will select variables that they believe that are relevant to answer a specific question. This characterization of input data might be biased and lead to potentially inequitable outputs or be constitutive of a direct discrimination based on a protected characteristic. The use of ‘race’ as a relevant category in medicine is a striking example of that risk. The absence of intent to discriminate is irrelevant.

First of all, it seems important to make a distinction between disaggregation of data based on protected characteristics in order to evaluate the efficiency of health policies on disadvantaged groups and the use of the same category as relevant variable for clinical purposes. While the first is heavily encouraged by human rights bodies in order to achieve effective rights, including in healthcare,^97^ the second will be regarded as suspicious. In Europe, the use of ethnic-racial data or on disability served eugenic purposes and the ideology of inferiority of certain populations. The program T4, which led doctors to renounce to the Hippocratic Oath to exterminate disabled persons explains why the use of such data is extremely sensitive and suspicious. The EU General Data Protection Regulation (GDPR) prohibits the collection of this ‘sensitive’ data, with several exceptions including in medicine and public health.^98^ Several countries prohibit the collection of such data and predictive algorithms on transplantation do not refer to race as a relevant criterion.^99^ In the United States, in contrast, data are not only disaggregated on the basis of multiple protected characteristics including race/ethnicity in order to improve public health and potentially detect health discrimination. ‘Race’ is recurrently selected as a relevant variable in the design of predictive algorithms evaluating the risks of chances of a successful transplantation.^100^ The selection of this variable can be questioned as such, ‘race’ being a social construct.^101^ Researches on human genome have challenged the classification of the population into five races and demonstrated that a small minority of alleles were specific to one geographical region.^102^ Thus, the use of ‘race’ as a variable might be based on the erroneous making of a causal link between certain medical factors and ‘race’ leading to generalization/overinclusion and inaccuracy. For example, the association between the creatinine serum rate and Black ‘race’ because of the muscularity of back people has been extremely debated.^103^ Moreover, the social nature of this factor explains why self-identification of patients to a certain ‘race’ might result in poor utility for clinical purposes, although these data can have another social usefulness.^104^

From a non-discrimination perspective, algorithmic predictions and classifications on this basis could be analyzed as potential direct discrimination on a racial ground or in association with a national minority (unless proven to be justified—according to the ECtHR’s jurisprudence). Besides, in the medical area where variables are not only socially useful, ML generalizations and predictions made on the basis of an association to a protected ground according to self-identification raises the following issue: in order to be protected from direct discrimination on the ground or race/ethnicity, does the person have to effectively belong to the protected group? Or is it sufficient that the decision is based on an assumption? Although this section focus is on the impact of developers’ data pre-processing, the risk of algorithmic discrimination based on—possibly inaccurate—perceptions or assumptions goes beyond the issue of self-determination and applies when algorithms erroneously learn by themselves individuals’ belonging to protected groups. Such risks are not clearly regulated in Europe. As previously stated, discrimination by association to a protected ground has been applied only when another person, herself part of a protected group, was involved. Discrimination by perception or assumption has only been recognized so far in case of indirect discrimination by the CJEU.^105^ When ‘race’ is selected as a variable, however, and disadvantaging one particular group, direct discrimination is more likely to apply. Nevertheless, despite the openness of the European Commission to consider such form of discrimination as a direct discrimination,^106^ Courts have not interpreted direct discrimination as encapsulating discrimination by wrong perceptions or assumptions. Thus, the protection of individuals in these circumstances by non-discrimination law remains unclear.

In addition to being suspicious and biologically irrelevant, the selection of race as a variable for organ transplantation can lead to inequitable outcomes for patients, especially some of the most vulnerable ones. Recent developments in kidney allocation have questioned the use of donor’s ‘race’ as a relevant criterion. Traditionally, the donor variable ‘African American’ has been considered a risk factor in kidney donation, which led the Kidney Donor Risk Index (KDRI) to place a higher penalty to kidneys from African-American deceased donors. For human transplantation, authors have shown that the substitution of race by APOL1 genotyping in the Kidney Donor Profile Index (KDPI)-based allocation led to more objective and better predictions.^107^ More precisely, the substitution has shown that long-term outcomes of kidney transplantation from African American donors with no or one APOL1 RRv were equivalent as European American deceased donors, questioning the relevance of a donor’s race as a relevant variable in risk prediction algorithms. In short, while the race factor conducted to exclude some organs, the genotype factor allows to offer more organs for transplantation and/or with a better utility. Providing that kidneys from deceased African-American donors are more likely to be transplanted to African-American patients because of medical factors (HLA, blood type), this variable would better serve the needs of a vulnerable group in access to organ transplantation: African-American patients.^108^

Although arguably more accurate, the substitution of ‘race’ by APOL1 should not lead to disregard other relevant factors of a successful transplant, including the socio-determinants of health.^109^ On another hand, the selection of this variable raises important ethical and legal issues related to the consent of living donors to retrieve genetic data information.^110^ In this regard, a recent study suggests the interest of African-American adults in genetic testing for living kidney donation.^111^ In Europe, the Oviedo Convention regulates the recourse to predictive genetic tests and prohibits ‘any form of discrimination against a person on grounds of his or her genetic heritage’.^112^ The substitution of ‘racial’ data by genetic data should not lead to another type of discrimination.

#### III.A.3. Conclusive observations on addressing the data input challenge

Addressing the inequitable outputs due to lack of and/or use of inadequate data would entail the adoption of relevant domestic and transnational frameworks to collect and use specific and appropriate data as encouraged by the CESCR^113^ and to prevent private actors to propagate the biases resulting from the data. Among the ongoing initiatives of the EU to strengthen the internal market for data, the proposal for a Regulation on the European Health Data Space may have the potential to facilitate the exchange of data between EU countries and to remedy the lack of data and its consequences on equitable access in Europe.^114^ In parallel, the EU Proposal of AI Act should contribute to prevent these risks, including for people in situation of socio-economic vulnerability.^115^ The Proposal’s current Article 10 imposes appropriate data governance and management practices in, i.e. data collection processes, data preparation, and formulation of assumptions which would apply to the above-mentioned issue of feature selection.^116^ The processing of sensitive data is submitted to strict conditions and necessitates appropriate safeguards to protect fundamental rights.^117^ The Act also imposes the relevance and representativeness of data sets, although it does not define these concepts, and requires a continuous monitoring. When it comes to the risk of data inadequacy, ‘[t]raining, validation and testing data sets shall take into account, to the extent required by the intended purpose, the characteristics or elements that are particular to the specific geographical, behavioral or functional setting within which the high-risk AI system is intended to be used’.^118^ Importantly, while the core provisions of the original draft did not include non-discrimination, the compromise text amended Article 10 by requiring appropriate governance in view of possible biases ‘that are likely to […] lead to discrimination prohibited by Union law’.^119^ The draft regulation does not, however, integrate a transversal and interdependent approach to fundamental rights. For example, data collection should be culturally appropriate and promote the participation of targeted populations. Inclusion is a necessary step to achieve equity in access to health resources.

### III.B. Algorithmic Architecture and the Risk to Exacerbate Vulnerability

The design of the algorithms on organ transplantation can also threaten equitable access to organs. This concerns both non-learning algorithms for organ allocation and ML algorithms. In the first case, risks may arise when developers—the ‘architects’ of organ allocation algorithms, need to define the algorithmic ‘target’,^120^ ie determine who is the good candidate for transplant, and choose a relevant type of algorithm.^121^ The development of the organ allocation algorithm embodies, crystallizes, and extends the public health debates related to the principles of allocation of scarce health resources, and the balance between utility and equality. Although state authorities have discretion in this divisive issue of scarce resource allocation, human rights law imposes a transparent process to achieve equitable access,^122^ with due account for collective interests and individual rights and an immediate obligation not to discriminate.

Members of the transplant community have been using algorithms for matching donors and recipients at a national or international level for decades. When defining what makes a good transplant candidate, the algorithm ‘architect’ has constraints and can make decisions that may impact equitable access to organs. The following sections discuss the determination and incorporation of criteria for organ allocation into the algorithms, whether established by the government or specific policies (thus appearing as *normative constraints*) or, in the absence of such criteria, designed by developers themselves. While the first subsection deals with allocation of organs from deceased donors, traditionally based on the principle of distributive justice (1), the second section investigates the matching of living kidney donors and recipients in kidney exchange programs (2). Both subsections show the risks of imbalance between utility and equality, or even discrimination, in algorithmic design. The third subsection provides concluding observations on addressing these multiple risks (3).

#### III.B.1. Allocation of deceased donors’ organs: utility over equality in algorithmic organ allocation?

Due to cold ischemia, the time factor is important in organ procurement from deceased donors. Algorithms help to cope with this medical constraint by integrating and crossing a multiplicity of factors allowing to identify the best potential recipients in a short time. In order to determine who will be the best candidate(s) for transplant of a specific organ, algorithms rely on criteria of medical compatibility and point score systems, attributing different amounts of points to various features identified by policy guidelines or other medical factors. In many cases indeed, the selection of relevant features defining a good or best transplant candidate is an application of public health and ethical norms, guidelines, or standards on fair allocation of scarce health resources. However, data scientists have room to determine allocation rules. This section elaborates on some early stages of algorithmic planning and development, showing that potential threats to equitable access to organs—such as defined in Section II—may find their origin in the very definition of the objective of the algorithm and the following algorithmic classifications.^123^

Most transplanted organs come from deceased donors. Organ recovery from a deceased person can only take place if consent or authorization by the deceased and/or her relatives has been retrieved.^124^ It is up to the authorities to determine how to allocate the harvested organs according to public health needs. Indeed, unlike living organ donation, which is most of the time directed to a specific recipient,^125^ deceased organ donations are mainly allocated according to collective values, starting with the principle of justice and following national or international waiting lists when countries collaborate. In the Eurotransplant system for instance, the ETKAS program (Eurotransplant Kidney Allocation System) for donors below 65 years old integrates different allocation factors, including collective values and interests. First, it relies on medical factors: the donor and recipient characteristics [HLA, Mismatch Probability (MMP), high urgency, kidney after liver transplant].^126^ Second, the algorithm also incorporates the principle of justice and equitable access between individuals and regions or countries by relying on the time spent on the waiting list and by the incorporation of a point system establishing a balance between member countries and regions. Third, Eurotransplant takes into account the vulnerability of certain patients by taking positive action by awarding two types of bonus to paediatric patients.^127^ Fourth, the algorithm considers the geographical distance between the donor center and the transplant center, which reflects utility and pragmatism (due to ischemia). Fifth and finally, it is worth observing that except Germany, other member countries of Eurotransplant give a bonus for patients having donated one of their kidneys before.^128^ The integration of this moral/social worth feature reflects the choice to valorize an original altruistic action by applying reciprocity. Some defend even further social worth or individual responsibility as relevant criteria to determine who can get an organ.^129^ The collective values and interests defining prioritization in access to organs differ depending on the regions and the period. They are heavily impacted by political choices. In the early stages of dialysis in the United States for instance, doctors decided that only patients living in Washington would have access to this technology because the State of Washington had financed its development.^130^ The different levels of investment (in research, infrastructures, etc.) in transplantation still contribute to explain today the disparities between regions in transplantation rates. These disparities are also related to different conceptions of *equitable access* and the level at which it should be achieved when increasing numbers of people on waiting lists foster competition for self-sufficiency in organs between regions on a national scene, and countries on the international scene.^131^

Even when prioritization is not based on moral virtue but on factors of medical relevance of apparent neutrality, such policies can infringe equitable access to organ transplants by having negative impacts on certain groups. Efficiency-based algorithms may result in an aggravation of the state of vulnerability of certain patients. For example, it has been shown in the United States that a purely efficiency-based algorithm targeting the greatest increase in quality-adjusted life expectancy would result in the assignment of lower priorities to African-American patients because of their higher risk of graft failure.^132^ While relying on an apparently neutral target, the algorithm disfavored a group of people protected by the law. Besides, in 2011, the OPTN proposed a policy to establish a kidney profile index (KDPI) and to reserve the best 20 per cent of kidneys to the 20 per cent of the candidates with the highest estimated post-transplant survival (EPTS) and to allocate the remaining 80 per cent of kidneys by age-matching whereby candidates within 15 years of the donor are given priority.^133^ On top of the risk to indirectly discriminate certain groups of patients, the criterion of longevity for organ allocation strongly correlates with age and may lead ML algorithms to systematically exclude the elderly from eligible transplantable candidates to the extent that it could be analyzed in terms of direct discrimination. Unlike the Supreme Court of the United States, which does not consider age as a suspect class under the equal protection clause,^134^ European Human rights law recognizes the elderly as protected category. The EU Charter of fundamental rights proclaims the equality before the law^135^ and includes ‘age’ in its non-limitative list of protected groups against discrimination.^136^ At the Council of Europe level, the Committee of Ministers of the Council of Europe adopted a recommendation in 2014 reminding that ‘[o]lder persons shall enjoy their rights and freedoms without discrimination on any grounds, including age and recommending Member States to make explicit reference to “age” in their national anti-discrimination legislation’.^137^ In addition, the issue of equitable access to healthcare for the elderly is among the planned actions of the strategic plan for 2020–2025.^138^ Beyond the special case of transplantation, the elderly lack of information and suffer from ‘age-based rationing, priority-setting, and poorly founded concerns regarding their capacity to make healthcare decisions’.^139^ In a context of scarcity, the optimization of organ allocation is a legitimate purpose that will meet without any doubt the first part of the test under non-discrimination law. There are different approaches to the consideration of age in the allocation of health care resources.^140^ However, one can wonder if the mathematical assessment and balance of years of life is more acceptable in a distributive justice system than judgments on individuals’ social worth. Against the trend to prioritize young patients under ‘fair innings’ or ‘numerical minority’ arguments, some authors have argued that such an approach could ignore other determinants of medical well-being and undermine equality between patients, by failing to individualize them.^141^ The treatment of the elderly is a pressing issue, since one of the challenges for health law in Europe is population aging and ‘responding to the ageing of the population is a policy priority for the EU’.^142^ At first glance, the level of correlation/causality between the target and age might be so tight that it could meet two of the requirements of direct discrimination: (i) a differential treatment in comparison with other patients and (ii) based on age, a protected ground under European law. However, a central issue here would be the test of comparability of situations, bearing in mind Courts’ discretion in this regard (*supra*). In the terms of the ECtHR, the difference of treatment should be between ‘persons in an analogous or relevantly similar situation’ in light of the subject-matter and purpose of the contested measure.^143^ On the one hand, if Courts were to find that the situations of older patients were relevantly similar to younger patients, then it would be harder for the authorities to prove that the contested measure was justified by external reasons to non-discrimination.^144^ On the other hand, if judges were to consider that the level of correlation between the protected characteristic—age—was not sufficiently narrow—because it affects other groups for instance, the situation could fall into indirect discrimination due the failure to take specific measures aiming at making effective the rights of members of a protected group. There would then be an application of apparently neutral measures with ‘disproportionately prejudicial effects’^145^ on individuals belonging to this group. As stated in Section II though, when it comes to health, there are multiple vulnerable groups and States are granted discretion in the management of scarce resources. Moreover, the elderly are a protected group but, as noted above, Courts do not apply the same rigorous standard of review than for other groups such as ethnic minorities. The scrutiny would certainly be on necessity and proportionality. The ECtHR would likely analyze whether the authorities had managed a fair balance between the rights of an elderly applicant and other interests including the utility imperative. In the US example, old people are excluded from the potential recipients of the best kidneys but will continue to be eligible for matching with other kidneys. In Europe, the existence of alternate programs for older recipients such as the specific Senior Program in the Eurotransplant model^146^ would likely exclude a finding of disproportion. In that respect, the disadvantage in qualitative transplantation does not appear disproportionate.

When combined with misconceptions or discriminatory assumptions on the good quality of life, the logic of utility sometimes leads to deny individuals with physical or mental disabilities access to the waiting list and to organ transplants.^147^ In the USA, discrimination in access to waiting list and transplant led the federal government to adopt the Charlotte Woodward Organ Transplant Discrimination Prevention Act in 2019.^148^ Although the above still holds true for persons with disabilities, they belong to a protected class to which international and European human rights law offer a higher protection. Therefore, a scrutiny of algorithmic outputs based on longevity matching would be stricter than for old people if they were prejudicial to persons with disabilities.

Finally, one last issue concerns the extent of positive measures at the algorithmic level to address inequities. Relying on socio-economic determinants of health, is there a right to algorithmic compensation for disparities in physical accessibility to transplantation that would imply to give more weight in algorithms in the same way as for medical necessity? Physical accessibility is directly linked to the rules of transplant allocation. As previously stated, available organs mainly come from deceased donors. For reasons related primarily to ischaemia, countries have adopted organ allocation policies that favor local allocation. Therefore, depending on respective hospitals’ transplantation activity, there is a correlation between regional (post-mortem) donation rates and regional transplant rates.^149^ Providing that there is often a correlation between geographical disparities and economic disparities and that discrimination on the basis of one’s economic situation is prohibited, States have a strong interest in taking positive measures to address inequities by adapting their allocation policies.

#### III.B.2. Matching living donors and recipients in kidney exchange programs: identifying the good match and the interplay with donor and physician subjectivity

The development of living donor kidney exchange programs may also, somewhat unexpectedly, raise some issues of equitable access. Living donor kidney transplantation is the preferred mode of treatment for patients suffering from end-stage kidney disease, since it allows for longer graft survival than transplantation of a deceased donor kidney.^150^ In most cases, living donations of kidneys do not involve the development of allocation algorithms since a person consent to give a kidney to a specific person she is usually bound with and her kidney is not put in a pool. However, following the examples of Switzerland and the Netherlands, several European countries have developed crossed or paired kidney donations between living donors and potential recipients.^151^ Concretely, it allows transplanting the kidney of living donor A to recipient B, when intended recipient A was not compatible with her/his donor A. In exchange, recipient A will receive the kidney from living donor B. Paired donations are efficient when they involve multiple pairs donor-recipient, facilitating multiple matching. Although at a different pace, European countries are progressively extending their kidney exchange programs.^152^ The number of pairs involved varies from jurisdiction to jurisdiction, such as the inclusion of non-directed donors in the chain (when such ‘altruistic’ donations are allowed). When several pairs are involved, countries may cooperate in different forms to find better matches. Scandiatransplant for instance has developed a transnational kidney exchange program between its members by merging the pools of pairs.^153^

Since the allocation of kidneys in the paired-donations programs is supposed to be based on exchange and reciprocity, the issue of equitable access to organs should not be relevant here, at least not directly. Yet, the management of paired-donations and the design of matching models—especially at a broader scale—may have an effect on equitable access to organs that I discuss below.

As with the allocation of organs from deceased donors, mathematical models are used to match donors and recipients, based on a variety of criteria including blood type, HLA data, clinical information, etc.^154^ One thing to consider is that unlike deceased donations though, which rely, most of the time,^155^ on the principle of distributive justice, living donations are mostly directed to a specific recipient.^156^ From the donor’s perspective, paired donations do not affect the fact that the consent to give a kidney is intrinsically linked to the therapeutic need of a designated recipient. Hence, one pertinent issue from an ethical and cultural perspective might be to determine if living donors in such chains have a right or interest to direct the kidney donation according to subjective criteria, thus impacting the allocation criteria of kidneys in those chains. Such possibility would introduce individual moralities in the allocation process, which are preeminent in classical living donations but that could potentially harm the interest of these exchange programs.^157^ It seems that the design of current kidney exchange programs overlooks the specificity of living donation but rather extends the above-mentioned discussions on utility and equality when it comes to determine what makes a best match, especially when multiple pairs are involved. As with deceased donations, this raises the issue of prioritization criteria for matching and, with that, who is to decide. Freedman et al.’s study on kidney exchange programs provides an example of a prioritization rule based on the highest number of matches in all circumstances.^158^ Again, maximizing the number of transplantations is a compelling interest. However, favoring utility over the medical disadvantages of certain patients (eg blood type O) who have fewer chances to find a suitable organ might result in the aggravation of their vulnerability if the utility choice is not compensated by positive actions in their benefit. Some European countries have chosen to encode this reality in order to improve equal access, by awarding in such programs a prioritization to blood-type-0 recipients for which the donor pool is the smallest and/ or prioritizing recipients according to low matching probability.^159^

From a substantive perspective, computer scientists might operate in a normative vacuum and search for criteria enabling them to develop pairing algorithms, through empirical analysis. In the above-mentioned study on kidney exchange model to align with human values for instance, Freedman et al. offered to ‘*elicit from human subjects a list of patients’ attributes they consider acceptable for the purpose of prioritizing patients in kidney exchanges*’.^160^ Their survey (100 participants) revealed that participants identified mostly three attributes categories to prioritize patients: age, health behavior (controllable by the patient), and general health (uncontrollable by the patient). To a lesser extent, the respondents also valorized patients who had dependents.^161^ The identification of human values is essential and a first step toward socially rooted algorithms. In fact, it may be argued to be an important aspect of the right to health which requires the cultural appropriateness of health services.^162^ Nevertheless, the measure of such values cannot only inspire from empirical analysis of individual moralities, potentially endorsing patients shaming and stigmatizing, but needs to rely on common ethical values and international and national legal standards.

Additionally, kidney exchange models may interact with equitable access to organs when, for pragmatic reasons, they integrate external (non-directed) kidneys in the chains, disrupting the logic of exchange and reciprocity between pairs. Indeed, there is a risk that one or more intended living donors withdraw their consent, potentially harming the compatible recipient. Such right to withdraw is protected by European instruments^163^ and is compensated by the utilitarian inclusion of kidney from deceased donors or ‘altruistic’ living donors in the chain. From the perspective of utility, this operation improves the efficient matching in these chains of living donors and recipients. It may be economically more interesting to encourage such programs early rather than having patients on dialysis. From a social justice perspective, the allocation of highly demanded organs to a chain instead of allocating them according to a waiting list based on fairly established criteria would need to be compensated by the reintegration in the chain of an equivalent kidney.

A last observation concerns human intervention during the deployment of the algorithm. Kidney exchange algorithms generate rankings between different solutions, enabling clinicians to decide in the end. In this regard, the physicians’ acceptance of kidney offers may play on exchange algorithms involving multiple pairs. In addition to the above-mentioned risks in the design of algorithms, collection and pre-processing of data for ML, or in the ethico-political definition of what makes a good candidate for transplant, biases and unequal outputs may also result from the physicians’ own personal biases^164^ and legitimate interests in securing the best transplant option for their patients.^165^ In the initial phases of kidney exchange models, where limited pairs were involved, the rejection of a kidney offer by a hospital was detrimental to the whole chain, with negative repercussions on transplantation rates.^166^ The enlargement of participants in these procedures increased the chances to propose alternate optimization matching in case of rejection. Hospitals Physicians still need to give their decision early in the process and the withhold of the easy to match pairs (with donors blood O for instance) remains a challenge from the perspective of vulnerable patients. However, this is not to suggest that doctors should encourage compatible pairs donor/recipient to participate in kidney exchange programs instead. European instruments are not as liberal as US law, living donations being considered with caution and non-directed donations being rather exceptional.

#### III.B.3. Concluding observations on rules to allocate organs

The risks of undermining equitable access to organ transplantation in the making of algorithms are often the consequence of broader policy choices and rules on priority-setting. European Human rights law only partially covers these risks, since state authorities are recognized an important margin in the balance and weighting of different interests. To take the example of disparities in physical accessibility to transplantation from deceased donors, authorities will need to assess the need for algorithmic compensation of disadvantage*.* Hypothetical ways to address this issue without facing the ischemia constraint might be to (i) granting more points in the algorithm to these patients when it comes to the allocation of organs from deceased donors in brain death, or in controlled cardiac death.^167^ Brain death is an alternate criterion of death endorsed by the international community, while controlled cardiac death follows a decision to withdraw life-supportive therapies (Maastricht III). Both types of post-mortem organ procurement allow for some organ preservation, giving doctors time to consider positive action for patients waiting for transplants who are usually disadvantaged because of where they live. (ii) To encourage living donation and to incorporate pairs in crossed donation algorithms. Potential recipients could receive financial help to travel to the transplantation center. Readjustments of this type are a matter of public health and ethics, but they should be based on a balance with other legitimate interests and account for social determinants of health in order not to exacerbate inequalities for the sake of good intentions.^168^

More generally, States must engage in a fair deliberative process, with due account for individual rights, including the right to equality and non-discrimination. Non-discrimination implies that all actors are aware of potential sources and forms of biases. A fair process in the algorithmic allocation of organs would also encourage States to promote patients and citizens’ participation in determining matching rules, within a certain frame and beyond the technical and medical constraints. One could imagine inclusive citizen consultations on algorithmic development, such as those that are sometimes held in the field of bioethics.^169^

### III.C. Machine Learning Deployment and the Risk of Currently or Potentially Unacceptable Correlations

Besides the collection and characterization of data by researchers, ML algorithms may produce inequitable or socially unacceptable outputs ‘themselves’, by making problematic correlations and detecting proxies to which European non-discrimination law only partially replies (1). Furthermore, ML may also favor the creation of algorithmic groups unprotected by European standards. States will need to determine whether correlations leading to these new groups are acceptable or not (2).

#### III.C.1. Socially unacceptable correlations and proxy discrimination

Discrimination or aggravation of vulnerability may result from the algorithmic planning and development, whatever the type of algorithm. When ML is used in particular, the learning process may generate biased outputs that this section focuses on. ML is used for organ allocation strategies but also for clinical prediction and decision support,^170^ prediction of waiting list mortality, prediction of graft survival or rejection, to analyze transplant biopsies, etc. ML models may analyze complex heterogeneous datasets and better handle multidimensional interactions between predictors.^171^ In contrast with classical programming, ML systems—including DL—are capable of generating rules that can later be applied to other datasets. Therefore, an inaccurate feature selection during data pre-processing such as described *supra* will likely generate [clinically] inaccurate rules. The irrelevance of retrospective/historical datasets in present-day clinical practice^172^ or the imbalance of the input data will affect the model performance to the detriment (for the latter) of underrepresented groups with rarer conditions.

However, ML algorithms may also produce discriminatory outputs in the absence of biases or inaccuracy in the data pre-processing. This is usually referred to as unequal ground truth. In ML, ground truth is considered to be ‘the best available approximation of reality, expressed in data’.^173^ Indeed, supervised ML algorithms establish correlations in large amounts of data, on the basis of which classifications or predictions of numeric outcomes are made. Providing that even strong, correlations are not causation, an inherent and structural issue of ML in regard to non-discrimination is the inevitable generalization and the lack of individualization. The risk of inaccurate association with a certain group exposes patients to discrimination by perception, or even multiple or intersectional discrimination that current European law cannot capture.^174^ Although human reasoning might fail to offer better solutions and ML improves patients’ health precisely because of these correlations with a high quantity of data, this issue is of particular concern due to the complexity and opacity of algorithmic systems.

Such correlations are even more suspicious when ML systems detect ‘proxies’.^175^ It is the case when, despite the absence of selection of protected characteristics as relevant features, the algorithm relies on variables correlated with membership in a protected group. Researchers frame this problem as ‘redundant encodings’.^176^ Blinding the algorithm to sensitive features may even aggravate the negative effects since the detection of discrimination becomes harder.^177^ A relevant example is the processing of residency data by predictive models, when it correlates to origin and ethnicity. As stated *supra*,^178^ locality is taken into account in deceased donors’ organ allocation algorithms because of the few hours of preservation of organs after their recovery. The development of ML based on such data will lead algorithms to make correlations on the basis of locality. However, this pragmatic criterion might hide, in a significant proportion, membership in protected classes, such as ethnicity, or strongly correlate with other types of vulnerability.

In order to mitigate these risks, computer scientists have developed computational techniques such as discrimination-aware data mining and fairness, accountability, and transparency in ML (FATML). Such techniques enable them to evaluate the differences in outcomes and measure equity and provide ‘reasonable ways to measure fairness’.^179^

They have inspired from non-discrimination law and privacy, including the GDPR.^180^ Nonetheless, there is a trade-off between utility and bias minimization in the algorithmic development,^181^ and the attempt to minimize socially unacceptable (yet exact) proxy discrimination will affect the algorithms’ predictive accuracy.^182^

European non-discrimination law only partially captures the risk of algorithmic discrimination, and in particular proxy discrimination. First, proxy discrimination will rarely enter within the scope of direct discrimination. The CJEU considers multiple factors in identifying discrimination on the basis of ethnicity, but these must be causally related to the protected characteristic in order to identify a direct discrimination.^183^ In case of proxy discrimination, the degree of overlap between a given proxy such as residency and the protected ground could be insufficient to establish the narrow link that the Court of justice requires for direct discrimination.^184^ Second, European non-discrimination law is currently unable to address in itself multiple and intersectional forms of vulnerabilities in health and transplantation. Such intersectional forms pre-exist in society and show up in the data, and the inability to address the complexity of their interactions would require a preventive approach.

#### III.C.2. Potentially unacceptable correlations: harms to non-legally protected algorithmic groups

Alongside the risk of making socially unacceptable correlations, ML may also identify new groups and generate new types of algorithmic differentiation that do not fit either the protected characteristics or the logic of non-discrimination law. For example, Dutch insurance companies have been shown to charge extra for car insurance when customers lived in apartments which numbering including a letter.^185^ Other groups such as ‘dog owners’, ‘single parents’ have been created by AI systems for purposes of advertising and personalized pricing.^186^ The use of ML and the diversification of the sources and nature of data, which also applies to health and transplantation, will call for an assessment, within comprehensible distinctions,^187^ between acceptable and unacceptable differentiations from the perspective of equitable access to transplantation. In this assessment, one challenging aspect is that new algorithmic groups are instable^188^ and deprived of social meaning.^189^

If we consider that such new groups may be the subject of unfair treatment, there are several obstacles to address this issue under the framework of this article. First, such grounds are unlikely to be interpreted, as such, as new protected grounds. Although protected grounds are most of the time non-exhaustive under non-discrimination law,^190^ leaving room to consider new and concomitant forms of vulnerabilities, their particular selection by the drafters of the treaties was a response to social or historical prejudices and oppressions.^191^ Similarly, European Courts have protected new groups of persons, on the same rationale. Hence, it would be surprising if Courts were to recognize algorithmic grounds as protected ‘other status’ or ‘such as’ groups historically or socially stigmatized. Second, assuming a contrario that some algorithmic groups were to be recognized as new protected grounds due to algorithmic stigmatization and prejudicial effects (under the category of indirect discrimination), the concrete analysis of discrimination, in particular the test of comparability between situations, might be complex in view of the intersecting characteristics composing the algorithmic groups. Third, for the above-mentioned reasons, addressing algorithmic stigmatization based on non-protected grounds would require to understand equitable access beyond the narrow borders of non-discrimination law and interpreting the principle of equality in the algorithmic context. However, as discussed in this paper, European human rights law recognizes that States are in a better position to assess and achieve substantive equality in the allocation of health resources, beyond their immediate obligation not to discriminate. In this regard, the AI Act Proposal’s distinction between the ‘value of equality’ and the ‘right to non-discrimination’ illustrates a theoretical barrier to tackle unfair algorithmic differentiations.^192^

#### III.C.3. Concluding observations on the correlations challenge in ML

A preventive approach would be needed to address the correlations challenge and proxy discrimination. In this respect, indirect discrimination can guide the planning and development of algorithms for organ allocation. By focusing on the effects of apparently neutral measures, it better addresses proxy discrimination. As shown *supra*, there are several procedural difficulties in the implementation of the test of indirect discrimination making it harder for patients to detect and claim that their rights have been violated. However, indirect discrimination encourages States to take positive action and account for disadvantaged groups. The inductive method of ML may contribute to identify structural issues, and more complex form of vulnerabilities policy-makers might have ignored, providing paths for actions. Upstream medicine can help to achieve equitable access by delimiting the scope of justified positive actions and address one of the challenges developers face: lack of knowledge on correlations with protected characteristics.^193^ If a person suspects that she has been discriminated against, the use of statistical data can also alleviate the difficulties of accessing evidence of indirect discrimination. The ECtHR has accepted the use of such data to determine the existence of indirect discrimination.^194^

When it comes to algorithmic (unprotected) groups, new theoretical approaches can fill the legal gap.^195^ From the perspective of legitimacy and sustainable algorithmic systems, the debate on acceptable and unacceptable groups should not remain the prerogative of experts alone but also involve the civil society. Within the framework of this article, a health and human rights approach could help in certain cases to interpret and adapt the principle of equality in the algorithmic context and to overcome the limits of non-discrimination law. For example, if an algorithm was generating a category of people based on their smoking or sport habits, leading to decisions harming their interests, a health and human rights reading could theoretically compensate for the absence of non-discrimination law protection for smoking/non-sporting people by providing a contextual reading of these new groups, linking to other factors of vulnerability, and avoiding the entrenchment of algorithmic stigma. This approach is compatible and should arguably be cumulated with other legal frameworks such as the right to algorithmic individualization protected by the GDPR.^196^

## IV. CONCLUSIONS: DELINEATING STRUCTURAL UPSTREAM REFORMS AND ALGORITHMIC DUE DILIGENCE IN FAVOR OF EQUITABLE ACCESS TO TRANSPLANTATION

The article has shown some of the risks of unfair outcomes in the design and use of algorithms for organ transplantation and the interplay with the European legal definition of equitable access to health resources. Such threats are rarely born with digitization, but digitization can amplify and systematize inequitable outputs.

The article completed more general research on algorithmic discrimination by concretely examining the difficulties to apply non-discrimination standards since on the one hand, current categories of discrimination do not fully reflect algorithmic reality and, on the other hand, access to biomedical resources inevitably rests on a balance of interests and values. Algorithms may perpetuate or produce disadvantageous effects for certain groups of people, but the associated risk of indirect discrimination will almost always meet the test of necessity and proportionality and is unlikely to exceed the wide state margin of appreciation in the allocation of scarce resources. In addition to the opacity of algorithmic systems, the lack of uniform approach in European legal instruments and jurisprudence may affect legal certainty and make litigation more complex.

In this regard, the clarification and adaptation of European non-discrimination standards would be desirable.^197^ Other frameworks such as privacy, briefly mentioned in this paper, can enrich the interpretation of fairness in law and contribute to the reaching of a more equitable access to organ transplantation. Although the EU Proposal of AI Act addresses certain of these issues, it is part of several EU initiatives based on market issues^198^ and should be complemented by an appropriate human rights-based instrument. The project to develop a proper international convention on AI and human rights at the level of the Council of Europe may fill the gap: it is the occasion to adopt a dedicated instrument and adjust the definition of equality and non-discrimination to the algorithmic context.^199^

The article also emphasized the importance of looking at the use of algorithms in organ transplantation in their context, rather than only focusing on ML discriminatory outputs. The above-mentioned threats to equitable access may emerge at different stages and involve different actors. Addressing such risks requires a preventive approach and will not be solved at the algorithmic level only. It will involve the development of a chain of duties, including long-term structural obligations and concrete immediate actions. International and European Human Rights Law, including the UN Guiding principles on the responsibilities of transnational corporations, can provide an internationally coherent framework to share and define responsibilities from the planning, to the development and selling/buying of algorithms.^200^ The future AI Act, which will apply to providers and users of medical devices in the EU,^201^ should fit into this transnational procedural framework. Its preventive approach can complete the redress-based approach of European non-discrimination law.

It seems important to delineate the scope of state obligations and the need of developers’ due diligence in order to achieve an equitable access to transplantation. In particular, due diligence involves human-rights impact assessments by private sector actors developing algorithms to be used for organ transplantation.^202^ States have obligations to identify and address barriers to equitable access to organ transplantation and can achieve this objective with the help of algorithms. In the ECtHR’ typology, such positive obligations are of a substantive and procedural nature. They may be of a structural nature.^203^

*First*, States need to encourage upstream medicine in order to assess the social determinants of health that may affect organ transplantation. Although the article principally discussed organ allocation algorithms, numerous persons in need of a transplant are not registered on national waiting lists. States need to address the reasons for the disparity in access to the waiting lists in the first place, the death rate while on a waiting list, and the disparities in access to organ transplants in a later phase. Algorithms may help to address the socio-economic determinants of health in the case of transplantation, by assessing risks of developing disease and need for transplant, looking at different communities’ living circumstances. As stated above, a gender perspective should be integrated by the disaggregation of health and socio-economic data according to sex.^204^

*Second*, and on the basis of the above, States should address disparities by determining the scope of positive action as normative constraints playing on the allocation algorithms while avoiding the creation of new imbalances. An example is the issue of algorithmic compensation to address disparities in access. Although non-discrimination law is not providing clear answers on the scope and extent of positive measures in that regard, a health and human rights suggests to further down what established policies already do in the allocation of organs from deceased donors. Such type of positive measures or ‘special measures’ to address inequalities have the advantage of being transparent and can be temporary as required by European law; the time for more structural reforms to produce effect. Indeed, adjustments at the allocation stage need to be constantly revaluated in order not to result in new inequalities, and a fair balance should be reached in addressing different sources of vulnerability. Besides, and as already mentioned, redistribution resulting in algorithmic compensation needs to account for studies on social determinants explaining disparities in access in a broader context.^205^ Regarding the use of the planning and development of ML, States should facilitate the collection and use of appropriate data by adapting their legislation to achieve equivalent quality predictions between patients. The EU AI Act will partly contribute to the achievement of this mission since it will be directly applicable in the EU Member States. Since developing ML with new datasets is costly, there is a compelling interest in ensuring the inclusiveness and appropriateness of data itself and collection methods in the first place. In the meantime, mitigating biases can limit disadvantage on certain groups.

*Third*, States need to foster education on ethics and human rights for data scientists as well as in medical schools.^206^ Interdisciplinary dialogue must be strengthened in order to effectively prevent the risk of potentially discriminatory algorithmic outputs.

## Funding

This research is part of the first UCPH Data+ project co-funded by the Faculty of Law and the Department of Computer Science DIKU at Copenhagen University.

## Footnotes

1 58,122 patients active on the waiting lists of EU Member States for kidney, liver, heart, lung, and pancreas only according to the data of the last Newsletter Transplant International: European Directorate for the Quality of Medicine Healthcare (EDQM) Newsletter Transplant International 2021, International Figures on Donation and Transplantation 2020, 25 (2021).

2 Such as defined by WHO as ‘the process that includes donor identification, evaluation, obtaining consent for donation, donor maintenance and retrieval of cells, tissues or organs’: see *Global Glossary of Terms and Definitions on Donation and Transplantation* (Geneva, 2009).

3 On top of other bilateral agreements. Eurotransplant gathers Austria, Belgium, Croatia, Germany, Hungary, Luxemburg, Netherlands, and Slovenia, Scandiatransplant gathers Denmark, Finland, Iceland, Norway, Sweden, and Estonia (associate member), and the SAT gathers France, Italy, Portugal, Spain, Switzerland, and Czechia (observer).

4 Directive 2010/53/EU of the European Parliament and of the Council of July 7, 2010 on standards of quality and safety of human organs intended for transplantation, OJ L 207, 6.8.2010, pp. 14–29. See also Commission implementing Directive 2012/25/EU of Oct. 9, 2012 laying down information procedures for the exchange, between Member States, of human organs intended for transplantation OJ L 275/28, 10.10.2012.

5
*Id*. Art. 7—see annex part 1 the minimum data set relates to: The establishment where the procurement takes place and other general data; Type of donor; Blood group; Gender; Cause of death; Date of death; Date of birth or estimated age; Weight; Height; Past or present history of IV drug abuse; Past or present history of malignant neoplasia; Present history of other transmissible disease; HIV; HCV; HBV tests; Basic information to evaluate the function of the donated organ. Annex Part B includes a list of complementary data.

6
*Id.* Art. 21.

7 For an overview of the use of algorithms in the field see Katie L. Connor et al, *The Future Role of Machine Learning in Clinical Transplantation*, 105 Transplantation (2021) 4, 723–735.

8 For instance to elaborate risk calculator on kidney allocation UdeM Nouvelles, *Attribuer le bon organe au bon moment: l’intelligence artificielle à la rescousse*, Apr. 24, 2017.

9 27 Member States in the EU since Brexit, and 46 Member States of the Council of Europe since Russia departed the organization due to the invasion of Ukraine.

10 Interactions are of political and legal nature. On the presumption of equivalent protection see ECtHR, Bosphorus Hava Yolları Turizm ve Ticaret Anonim Şirketi v. Ireland, No. 45036/98 (2005). Other types of interactions include cross-fertilization through the jurisprudence of the EU and the Council of Europe’s respective Courts. Besides, the EU shall accede to the ECHR.

11 Council of Europe, Committee on Bioethics (DH-BIO), Strategic Action Plan on Human Rights and technologies in Biomedicine (2020–2025), Nov. 19–21, 2019. Although the EU has a limited competence in health, see also Council Conclusions on common values and principles in European Union Health Systems, OJ C 146/1 22.06.2006, recalling ‘the overarching values of universality, access to good quality care, equity and solidarity’.

12
*Id*. (Committee on Bioethics (DH-BIO)) at 19–20.

13 Studies are numerous. For an overview in health, see WHO guidance, Ethics and Governance of Artificial Intelligence for Health (2021). See, eg Mathias Risse, *Human Rights and Artificial Intelligence: An urgently Needed Agenda*, 41 Human Rights Quarterly 1 (2019), Brent Mittelstadt et al. *The Ethics of Algorithms: Mapping the Debate*, Big Data & Society 1 (2016), Alessandro Mantelero, *AI and Big Data: A blueprint for a human rights, social and ethical impact assessment*, 34(4) Computer Law & Security Review 754 (2018) etc (some reference are used below).

14 European Commission, Proposal for a Regulation of the European Parliament and of the Council laying down harmonized rules on artificial intelligence (Artificial Intelligence Act) and amending certain Union legislative acts, COM/2021/206 final, Apr. 21, 2021; and Presidency compromise text, Nov. 29, 2021. Hereafter “Proposal of Artificial Intelligence Act”. Other instruments for health include the Medical Device Regulation, aiming to ‘to ensure the smooth functioning of the internal market as regards medical devices, taking as a base a high level of protection of health for patients and users’, (recital (2). The Regulation heightens requirements for post-market surveillance and medical device traceability. It applies to software of prediction and prognosis of disease (art. 2). This Regulation, based on the market and safety of products, is not relevant for our study on human rights approach to algorithms. For a comparative study (with US regulatory framework), see Timo Minssen et al., *Regulatory Responses to Medical Machine Learning*, 7(1) Journal of Law and Biosciences (2020), https://doi.org/10.1093/jlb/lsaa002 (accessed 9 June 2022).

15 Explanatory Report to the Convention on Human Rights and Biomedicine (ETS 164, Oviedo, 1997), at 25.

16 Additional Protocol to the Convention on Human Rights and Biomedicine concerning Transplantation of Organs and Tissues of Human Origin (ETS 186, Strasbourg, 2002), art. 3.

17 Explanatory Report to the Additional Protocol to the Convention on Human Rights and Biomedicine concerning Transplantation of Organs and Tissues of Human Origin (ETS 68, Strasbourg, 2002), at 43.

18 European Social Charter (ETS 35, Turin, 1961) and Revised European Social Charter (ETS 163, Strasbourg, 1996) Article 11. See also EU Charter of Fundamental Rights, OJ 2010 C 83/389, which protects a right to healthcare ‘under the conditions established by national laws and practice’ (art. 35).

19 International Covenant on Economic, Social, and Cultural Rights (UNTS 993, p. 3, 1966), Art. 12.

20 Un Committee on Economic, Social, and Cultural Rights (CESCR), *General Comment No. 14, The Right to the Highest Attainable Standard of Health* (Art. 12), at 12 (b).

21
*Id*., at 12. Although the General comment is not binding, it has interpretative value.

22 Pentiacova and others v. Moldova, No. 14462/03 (dec.) (2005), where the Court ‘notes that the applicants’ claim amounts to a call on public funds which, in view of the scarce resources, would have to be diverted from other worthy needs funded by the taxpayer. [blanck] While it is clearly desirable that everyone should have access to a full range of medical treatment, including life-saving medical procedures and drugs, the lack of resources means that there are, unfortunately, in the Contracting States many individuals who do not enjoy them, especially in cases of permanent and expensive treatment’.

23 See CESCR *General Comment No. 3: The Nature of States Parties’ Obligations (Art. 2, Para. 1, of the Covenant)*, at 9.

24 For example, ethical perceptions on *post mortem* donation, such as consent or authorization, death criteria, and removals on Maastricht III (controlled cardiac death), play on the availability of organs.

25 See Convention on Human Rights and Biomedicine (ETS 164, Oviedo, 1997), article 19(1); but see Additional Protocol to the Convention on Human Rights and Biomedicine concerning Transplantation of Organs and Tissues of Human Origin (ETS 68, Strasbourg, 2002), which nuances this principle. Concerning approval, see Additional Protocol concerning transplantation, art. 10; and Oviedo Convention, art. 19(2).

26 See for instance Additional Protocol concerning transplantation, art. 18 on the ‘respect for the human body’ after death. The ECtHR also found that the wish of the deceased fell within the scope of the right to private life (on disposal of ashes see the former European Commission’s findings in X v. Federal Republic of Germany, No. 8741/79 (1981); on the moving of a funeral urn and the wishes of the deceased see Elli Poluhas Dödsbo v. Sweden, No. 61564/00 (2006)). The deceased’s rights are also protected through the right of private and family life of the closest relatives (see in the context of contested recovery of tissues and organs Petrova v. Latvia, No. 4605/05 (2014) and Elberte v. Latvia, No. 61243/08 (2015)). For a comparative analysis on the nature and the holders of human rights after death (Europe, United States), see Audrey Lebret, Prélèvement d’organes et Droits Fondamentaux aux États-Unis et en Europe (Université Panthéon-Assas, 2019), at 515–640.

27 See CESCR *General Comment No. 3* at 8.

28 CESCR, *General Comment No. 14,* at 30.

29
*Id*. at 43 (a) and (e).

30 Treaty of Rome: Treaty Establishing the European Economic Community, Mar. 25, 1957, 298 U.N.T.S. 3, 4 Eur. Y.B. 412, Treaty of Amsterdam Amending the TEU, the Treaties Establishing the European Communities and Certain Related Acts, Oct. 2, 1997, 1997 O.J. (C340) 1, 37 I.L.M. 253, and above all, Treaty of Lisbon Amending the Treaty on European Union and the Treaty Establishing the European Community, Dec. 13, 2007, 2007 O.J. (C306) 1. See the EU Charter: Charter of Fundamental Rights of the European Union: 2010 O.J. (C83) 389. Proclaimed by the Commission, 7 December 2000. Proclamation and text at 2000 O.J. (C364) 1.

31 Proposal of Artificial intelligence Act. The legal basis for the Regulation on the right to personal data protection and the internal market.

32 See Council Directive 2000/78/EC of Nov. 27, 2000 establishing a general framework for equal treatment in employment and occupation (‘Employment Equality Directive’) COM(2014) 2 final (014).

33 Council Directive 2004/113/EC of Dec. 13, 2004 implementing the principle of equal treatment between men and women in the access to and supply of goods and services. The CJEC (former CJEU) established that health care was a ‘**service**’ under EC law in Cases C-286/82 and C-26/83, Luisi and Carbone v. Ministero del Tesoro, 1984 E.C.R. 377,403. The Court affirmed the applicability of the freedom of movement principles of the EC to social security systems in cases *Kohll* et *Decker from* 28 Avril 1998: *C*ase C-120/95, Decker v. Caisse de Maladie des Employds Privés, 1998 E.C.R. 1-1831 and Case C-158/96 *Kohll c. Union des caisses de maladie*, 1998 E.C.R. 1-1931. The exchange of harvested organs on the EU market makes also applicable the freedom of **goods**.

34 Council Directive 2000/43/EC of 29 June 2000 implementing the principle of equal treatment between persons irrespective of racial or ethnic origin, OJ L 180, 19/07/2000 P. 0022—0026 Art. 3(1(e)).

35 Scandiatransplant (A part from Finland which ratified the treaty in 2004, nor Estonia, Norway, Denmark, Sweden, or Iceland are parties to that date). Eurotransplant Croatia ratified in 2003, Luxembourg in 2006, the Netherlands 2004, Solvenia in 2010 (nor Belgium, nor Hungary, Germany). For the state of ratifications of the Protocol, see https://www.coe.int/en/web/conventions/full-list?module=signatures-by-treaty&treatynum=177 (accessed June 6, 2022)

36 European Social Charter, art. 13(4) and art. E.

37 Glor v. Switzerland, No. 13444/04 (2009) at 80; G.N. and Others v. Italy, No. 43134/05 (2009) at 126; Kiyutin v. Russia, No. 2700/10 (2011) at 57.

38 For a detailed overview, see Guide on Article 14 of the European Convention on Human Rights and on Article 1 of Protocol No. 12 to the Convention- Prohibition of discrimination (2021).

39 The Court has stressed that discrimination based on sexual orientation was ‘as serious as discrimination based on “race, origin or colour”’ (Vejdeland and Others v. Sweden, No. 1813/07 (2012) at 55).

40 EU Charter of Fundamental Rights, art. 21.

41 The European Committee of Social rights monitors the implementation of the Charter in the Council of Europe through national reports and a mechanism of collective complaints. Although its decisions and conclusions are not directly enforceable in the domestic legal systems, they play an essential role in the interpretation of social rights in Europe. For more on the justiciability of social rights in Europe and the role of the European Committee of Social Rights see Philp Alston, *Assessing the Strengths and Weaknesses of the European Social Charter's Supervisory System*, *in*  Social Rights in Europe 45–68 Gráinne De Búrca and Bruno de Witte (ed.) (OUP 2005).

42 For the ECtHR, see in particular Biao v. Denmark, No. 38590/10 at 89 (2016).

43 In EU law, intent is irrelevant for both direct and indirect discrimination: ‘proving a case of direct discrimination requires neither showing that the perpetrator was conscious of the discrimination nor showing that he or she intended to discriminate’, Janneke Gerards and Raphaëlle Xenidis (European network of legal experts in gender equality and non-discrimination), *Algorithmic Discrimination in Europe: Challenges and Opportunities for Gender Equality and Non-Discrimination Law* (Report for the European Commission, 2021) at 67. Intent is also irrelevant for indirect discrimination in the case law of the ECtHR, and also implicitly irrelevant in case of—at least—racial discrimination, regardless of its form: see ECtHR, D.H. and others v. The Czech Republic, No. 57325/00 (2007) at 176: ‘The Court has also held that no difference in treatment which is based exclusively or to a decisive extent on a person’s ethnic origin is capable of being objectively justified in a contemporary democratic society built on the principles of pluralism and respect for different cultures’. For an interpretation in that sense see the concurring opinion of Judge Pinto de Albuquerque in Biao v. Denmark at 7.

44 CJEU, Case C83/14, CHEZ Razpredelenie Bulgaria AD v. Komisia za zashtita ot diskriminatsia, Judgement of July 16, 2015,ECLI:EU:C:2015:480. at 56 (on racial equality and discrimination on the basis of Roma origin). See also CJEU, Case C-303/06, S. Coleman v. Attridge Law, Steve Law, [2008] I-05603 (discrimination on grounds of disability).

45 CJEU, CHEZ at 49 and 56, to read in conjunction with J. Kolott, conclusions 2015 case CHEZ at 57 and following. At the ECtHR, see for example Guberina v. Croatia, No.23682/13 (2016), in which the domestic authorities failed to take into account the needs of a child with disabilities when they determined his father’s eligibility for tax relief on the purchase of adapted property. The Court found that the applicant had been subject to discrimination on the ground of disability on the account of his child disability.

46
Manuela Brillat, Le principe de non-discrimination à l’épreuve des rapports entre les droits européens 123 (2015).

47 ‘Declaring that no new category of discrimination resulting from the combination of more than one of those grounds […] may be found to exist where discrimination on the basis of those grounds taken in isolation has not been stablished’. Case C-443/15 *David L. Parris v Trinity College Dublin and Others* (2016), at 80.

48 Iyiola Solanke, *The Anti-stigma Principle-centrealising Intersectionality in the Theory of Anti-discrimination Law*, in Actes du Colloque Multiplication des critères de discrimination—Enjeux, effets et perspectives (Défenseur des droits de la République française, Mission de recherché Droit & Justice), at 146.

49
*Id.* at 147. The concept had a procedural and substantive interest in the US where it emerged since it allowed applicants to be recognized as victims although no discrimination would have been found on respective grounds taken in isolation (the single-axis approach): *see* in particular US District Court for the Eastern District of Missouri, May 4, 1976, Emma DeGraffenreid et al. v. General motors assembly division, St Louis, a corporation, et al., N°75-487 C (3), 413 F. Supp. 142 (ED.Mo.1976). See Kimberle Creenshaw, *Demarginalizing the Intersection of Race and Sex: A Black Feminist Critique of Antidiscrimination Doctrine, Feminist Theory and Antiracist Politics*, 1 The University of Chicago Legal Forum 139 (1989).

50 See Case C-443/15 *David L. Parris v Trinity College Dublin and Others* (2016).

51 See for example BS v. Spain, No. 47159/08 (2012) in which the Court considered that the authorities had failed to take into account the applicant’s particular vulnerability as an African woman working as a prostitute (at 62) and found a violation of Article 14 in conjunction with Article 3; see also Carvalho Pinto de Sousa Morais v. Portugal, No. 17484/15 (2017) on sex and age.

52 Kathy Davis*, Intersectionality as buzzword—A sociology of science perspective on what makes a feminist theory successful*, 9(1) Feminist theory 67 (2008).

53
Ludovic Hennebel and Hélène Tigroudja, Traité de droit international des droits de l’homme 764 (2018).

54 Concerning the access to goods and services in healthcare, the preamble of EU Directive 2004/113/EC specifies for instance that ‘differences between men and women in the provision of healthcare services, which result from the physical differences between men and women, *do not relate to comparable situations* and therefore, do not constitute discrimination’. Directive 2004/113/EC, preamble at 12 (emphasis mine).

55 See also Samantha Besson, *Evolutions on Non-Discrimination Law within the ECHR and ESC Systems: It Takes Two to Tango in the Council of Europe*, 60 AM. J. COMP. L. 147 at 167. See the definitions of direct and indirect discrimination in EU relevant directives. These directives contain a very a few and restrictive exceptions: for occupational requirements (Council Directive 2000/43/EC of 29 June 2000 implementing the principle of equal treatment between persons irrespective of racial or ethnic origin *Official Journal L* 180, 19/07/2000 P. 0022-0026), see also (although not applicable here): Council Directive 2000/78/EC of 27 November 2000 establishing a general framework for equal treatment in employment and occupation *OJ L* 303, 2.12.2000, pp. 16–22*,* Council Directive 2000/78/EC of 27 November 2000 establishing a general framework for equal treatment in employment and occupation contains exception on the basis of age and using the framing of the ECtHR ‘objectively and reasonably justified by a legitimate aim’, art. 6, Directive 2006/54/EC of the European Parliament and of the Council of July 5, 2006 on the implementation of the principle of equal opportunities and equal treatment of men and women in matters of employment and occupation (recast), art. 14.

56 Directive 2004/113/EC, art. 4(5) stating that the ‘Directive shall not preclude differences in treatment, if the provision of the goods and services exclusively or primarily to members of one sex is justified by a legitimate aim and the means of achieving that aim are appropriate and necessary’: see also art. 5(1).

57 In the wording of the ECtHR, ‘appropriate and necessary’ in the wording of EU Directive 2004/113/EC.

58 The CJEU has been a driver on positive actions, to differentiate with affirmative action in the US. On substantive equality at the EU level see Marc de Vos, *The European Court of Justice and the March Towards Substantive Equality in European Union Anti-discrimination Law,* 20(1) International Journal of Discrimination and the Law 62 (2020).

59 See Thlimmenos v. Greece, No. 34369/97, at 44 (2000).

60 The ECtHR relied in international instruments such as the UN Convention on the Rights of Persons with Disabilities (CRPD) to affirm that there was a European and worldwide consensus on the need to protect persons with disabilities from discrimination, which included the obligation to ensure ‘reasonable accommodation’ see Çam v. Turkey, No 51500/08, at 65-67 (2016); G.L. v. Italy, No 59751/15, at 60-66 (2020). In that particular context, the Court stated that ‘discrimination on grounds of disability also covers refusal to make reasonable accommodation’ (Çam v. Turkey at 67). The EU is part of CRPD, such as all Member States of the Council of Europe (except Liechtenstein to this date) so it is a source of law in itself in Europe. Relevant provisions are art. 2, 3(2) and 5(3).

61 European Committee of Social Rights, Autism-Europe v. France, decision (merits), No 13/2002, at 53 (2003), finding that States are required to take legal and practical actions to give full effects to the rights contained in the Charter and, observing a ‘a chronic shortage of care and support facilities for autistic adults’, that France had failed in fulfilling its obligations.

62
Brillat, Le principe de non-discrimination... at 123, or as, called by Jean-Pierre Marguénaud and Jean Mouly a ‘super positive obligation’ (*Id*.).

63 Autism-Europe v. France at 53. In alignment with the UN CESCR, *General Comment No. 14*.

64 The jurisprudence is not homogeneous on the obligations to take measures to correct inequalities. Under certain circumstances, the ECtHR has identified violations of the ECHR when state authorities had failed to take measures to redress factual differences (see for example Thlimmenos v. Greece, No. 34369/97 (2000)). However, the interplay of positive measures with the margin of appreciation is variable (when these rights are economic and social in nature, this margin may complicate the identification of a positive obligation). Besides, in practice, the Court recurrently avoids the examination of non-discrimination claims (excluding de facto an analysis of a potential positive obligation deriving from the prohibition of discrimination) when it already found a violation of another article of the ECHR. See for instance the jurisprudence on Roma communities, the Court addresses these inequalities as part of its assessment of article 8 (right to private and family life) and is usually reluctant to examine claims of unequal access or unequal treatment as such (as part of article 14), see in particular Hudorovič and others v. Slovenia, App. Nos. 24816/14 and 25140/14 (2020) (finding that article 8 may impose States to take measures to provide access to safe drinking water and sanitation, but refusing to examine the non-discrimination aspect in spite of acknowledging the vulnerability of Roma communities in this regard). On the avoidance strategy on bioethical issues (reproductive claims), see also Evans v. United Kingdom, App. No 6339/05 (2007), at 93–96 (concerning the woman applicant’s right to become a genetic parent (v. the right of her former partner not to become a parent), in which the Court found that there was no separate question based on the combination of articles 8 and 14), and S.H. and others v. Austria, No. 57813/03 (2011) at 120, (concerning two couples’ access to IVF) in which the Grand Chamber did not find necessary to proceed to the analysis of non-discrimination claims.

65 J.D. and A. v. the United Kingdom, Nos. 32949/17 and 34614/17, at 84 (2019), Napotnik v. Romania, No. 33139/13, at 73 (2020).

66 J.D. and A. v. the United Kingdom, at 85.

67 EU Agency for Fundamental Rights (FRA), Handbook on European non-discrimination law (2018) https://fra.europa.eu/en/publication/2018/handbook-european-non-discrimination-law-2018-edition, at 41–42 (accessed 9 June 2022).. The ECtHR was not much confronted to the issue of special measures, but see Wintersberger v Austria where if finds that the positive discrimination in favor of persons with disabilities was justified and that the valid applicant’s request against that measure was inadmissible. For a definition of “special measures” to achieve racial equality, see Committee on the Elimination of Racial Discrimination (CERD), General Comment No. 32, *The Meaning and Scope of Special Measures in the International Convention on the Elimination of Racial Discrimination* (2009). Special measures should not lead to the maintenance of separate rights between groups, they shall not be continued after the objectives for which they have been taken have been achieved.

68 See in particular Sofia Gruskin and Norman Daniels, *Process is the Point: Justice and Human Rights: Priority Setting and Fair Deliberative Process* 98(9) American Journal of Public Health 1573 (2008). From a theoretical perspective, Human Rights Law is not to be confused with theories of justice despite their interactions. International Human Rights law is shaped by pluralistic conceptions of justice as reminded in Hennebel and Tigroudja, Traité de droit international des droits de l'homme at 24. The jurisprudence of the ECtHR demonstrates an influence of sociological theories, through the quest of a fair balance of interests and consensus rather than providing general and absolute solutions. This explains why European human rights law does not impose a uniform definition of equitable access and priority setting.

69 On the interactions between health and Human Rights and the benefits of a cross-reading of these fields, see especially J M. Mann et al., *Health and Human Rights*, 1(1) Health and Human Rights Journal 6 (1994). On the social determinants of health, see WHO reports at https://www.who.int/health-topics/social-determinants-of-health#tab=tab_1 (accessed 9 June 2022).

70 Which the authors frame as ‘proportionate universalism’: Michael Marmot et al., Fair Society, Healthy Lives: Strategic Review of Health Inequalities in England Post-2010 (the Marmot Review) 15 (2010).

71
*General Comment No 14*, at 3–4.

^72^
Although much research is still needed, North-American studies are more numerous than European ones. Studies suggest that patients living in rural areas had lower rate of waitlisting and transplant despite equivalent outcomes following transplant see David A. Axelrod et al., *Rates of Solid-Organ Wait-Listing, Transplantation, and Survival Among Residents of Rural and Urban Areas,* 299(2) JAMA 202*–*7 (2008); more broadly see Valerie A Luyckx et al., *Sustainable Development Goals Relevant to Kidney Health: An Update on Progress*, 17(1) Nat Rev Nephrol 15 (2021), Christine Park et al. *A Scoping Review of Inequities in Access to Organ Transplant in the United States*, 21 International Journal for Equity in Health 22 (2022). See also Osvald Nitski et al*., Long-term Mortality Risk Stratification of Liver Transplant Recipients: Real-time Application of Deep Learning Algorithms on Longitudinal Data*, 3(5) The Lancet Digital Health E295 (2021), showing that recipient education level along with the primary payment method in the USA are among the main predictive variables for post-liver transplantation mortality. For European studies see Jérôme Harambat et al., *Likelihood of Children with End-stage Kidney Disease in Europe to Live with a Functioning Kidney Transplant is Mainly Explained by Nonmedical Factors*, 29(3) Pediatr Nephrol 453 (2014) finding that the GDP was the most important determinant of international differences in the likelihood of children to live with a functioning kidney transplant, on racial disparities see Lidwien A Tjaden et al., *Racial Disparities in Access to and Outcomes of Kidney Transplantation in Children, Adolescents, and Young Adults: Results From the ESPN/ERA-EDTA (European Society of Pediatric Nephrology/European Renal Association-European Dialysis and Transplant Association) Registry*, Am J Kidney Dis (2016), finding less favorable outcomes for black and Asian patients. On the impact (or absence of) of socioeconomic deprivation on access see Lucy A Plumb et al., *Associations between Deprivation, Geographic Location, and Access to Pediatric Kidney Care in the United Kingdom*, 16(2) Clin J Am Soc Nephrol 194 (2021), Wahida Kihal-Talantikite et al., *Influence of Socio-Economic Inequalities on Access to Renal Transplantation and Survival of Patients with End-Stage Renal Disease*, Plos One (2016), showing that ‘in a mixed rural and urban French region, patients living in deprived or advantaged neighborhoods had the same chance to be placed on the waiting list and to undergo renal transplantation’.

73 See for instance Agnieszka Kisielska et al.*, The Impact of Socio-Demographic Factors on the Functioning of Liver Transplant Patients*, 19 Int. J. Environ. Res. Public Health 4230 (2022), investigating the levels of adherence and support after surgery and observing that ‘most support was received by women, people under 40 years of age, and those with secondary education’.

74 Eurotransplant Manual, version 5.5 (2016), Chapter 1, at 18.

75
Lebret, Prélèvement d'organes et droits fondamentaux..., at 79.

76 Park et al., *A scoping review of inequities in access to organ transplant in the United States*, at 14, Sabina De Geest and Kris Denhaerynck, ‘*Upstream Medicine’: Targeting Social Determinants of Health in Transplant Care*, 31 Transplant International 1083 (2018). Some initiatives in health in general contribute to address these issues, on the European Deprivation Index see for instance Élodie Guillaume et al., *Development of a Cross-cultural Deprivation Index in Five European Countrie*s, 70(5) Journal Epidemiology Community Health 493 (2015).

77 The acceptability of digital health systems goes beyond the absence of discrimination and requires consultation and participation of relevant populations as well as account for relevant cultural factors and values. These are, however, outside the scope of this paper.

78 This section focuses on data collection and inequitable outputs, but other connected and important issues apply to the data and play on the efficiency of ML. For example, Miller et al. have shown that large clinical data sets, in this case, UNOS data on cardiac transplantation, may lack the accuracy and granularity needed for ML methodologies to uncover unique associations, although it is a reason why we use ML instead of traditional statistical models: see P. Elliott Miller et al. *Predictive Abilities of Machine Learning Techniques may be Limited by Dataset Characteristics: Insights from the UNOS Database*, 25(6) Journal of Cardiac Failure 479 (2019).

79 Moritz Hardt, *How Big Data is Unfair*, https://medium.com/@mrtz/how-big-data-isunfair-9aa544d739de (accessed May 25, 2022), see also Alvin Rajkomar et al., *Ensuring Fairness in Machine Learning to Advance Health Equity,* 169 Ann Intern Med 866–72 (2018) frame it as “minority bias” at 868. In order to address the sample-size disparity and ensure that the patterns will hold true for more persons than those represented in the particular sample, Solon Barocas and Andrew D. Selbst, *Big Data’s Disparate Impact*, 104 California Law Review 671 (2016) consider that ‘the sample must be proportionally representative of the entire population, even though the sample, by definition, does not include every case’, at 686.

80 Christina Storm Mienna and Per Axelsson, *Somatic Health in the Indigenous Sami Population—A Systematic Review*, 78(1) International Journal of Circumpolar Health 1638195 (2019). For an analysis of this issue in Sweden and on Sámi perspectives on the ownership and governance of their health data see Per Axelsson and Christina Storm Mienna, *The Challenge of Indigenous Data in Sweden*, *in*  Indigenous Data sovereignty and Policy 99-100 (Maggie Walter et al. ed., 2021).

81
Caroline Criado Perez, Invisible Women- Exposing Data Bias in a World Designed for Men, 199–203 (2019).

82 Rieke Van der Graaf et al., *Fair Inclusion of Pregnant Women in Clinical Trials: An Integrated Scientific and Ethical Approach* (2018) 19 Trials 78, Daniel Schönberger, *Artificial Intelligence in Healthcare: A Critical Analysis of the Legal and Ethical Implications,* 27 International Journal of Law and Information Technology 171, at 180 (2019).

83
Criado Perez, Invisible women.

84 CESCR, *General comment No. 25 (2020) on science and economic, social and cultural rights (article 15 (1) (b), (2), (3) and (4) of the International Covenant on Economic, Social and Cultural Rights)*, E/C.12/GC/25, Mar. 6, 2020, at 30.

85 CESCR, *General Comment No. 14, *at 20. Specific concerns also apply to the implementation of the right to health for other groups such as indigenous communities. In particular ‘health services should be culturally appropriate, taking into account traditional preventive care, healing practices and medicines’ (at 27).

86 Silke Schicktanz et al., *Gender Issues in Living Organ Donation: Medical, Social and Ethical Aspects*, *in*  Sex & Gender in Biomedicine: Theories, Methodologies, Results 39 (Ineke Klinge and Claudia Wiesemann ed., 2011), see also Julien Hogan et al., *Gender Disparities in Access to Pediatric Renal Transplantation in Europe: Data From the ESPN/ERA-EDTA Registry*, in 16 American Journal of Transplantation 2097 (2016), Francesca Puoti et al., *Organ Transplantation and Gender Differences a Paradigmatic Example of Intertwining Between Biological and Sociocultural Determinants*, 7 Biology of Sex Differences 35 (2016).

87 A New Approach for Measuring Gender Disparity in Access to Renal Transplantation Waiting Lists; see also in France on CRISTAL cited in Valérie Pouliquen, *Inégalités sociales de santé en transplantation rénale* <https://tel.archives-ouvertes.fr/tel-02466514/document>, at 30 noting a delayed access to waiting list for women;

88 Hogan et al., *Gender Disparities in Access to Pediatric Renal Transplantation*.

89 See the recent study -with international participants- of Allison Tong et al., *Nephrologists’ Perspectives on Gender Dsparities in CKD and Dialysi*s, 7(3) Kidney International Reports 424 (2022).

90 Cheryl Kodjo, *Cultural Competence in Clinician Communication*, 30(2) Paediatr Rev 57 (2009).

91 Rajkomar et al., *Ensuring Fairness in Machine Learning...* at 868 frame this type of bias as ‘training–serving skew’.

92 W. Nicholson Price, *Medical AI and Contextual Bias*, 33(1) Harvard J.L. & Tech 65 (2019).

93
*Id*. at 67.

94 Johan Nilsson et al., *The International Heart Transplant Survival Algorithm (IHTSA): A New Model to Improve Organ Sharing and Surviva*l, 10(3) PLOS One (2015).

95 See for example a research team working on a DL algorithm in Québec using the data of Scientific registry of Transplant Recipients (SRTR), a US Registry. For a presentation of the project and broader discussions on big data and AI in organ donation, see Heloïse Cardinal, *The Development of a Clinical Decision Support Tool Integrating Machine Learning Approaches: Can We Improve Shared Decision Marking in Decision-making in Kidney Transplantation?*, Virtual conference June 17–18 2021, university of Ottawa, available at https://www.ottawahealthlaw.ca/odtconference (last accessed June 1, 2022), and specifying they will train their model on local datasets.

96 The importation of external in order to compensate lack of internal data on ethnical minorities may also raise broader issues of data colonialism when corporations develop in a predatory way their models with southern countries’ data. See a.o. Nick Couldry and Ulises A. Mejias, *Data Colonialism: Rethinking Big Data’s Relation to the Contemporary Subject*, 20(4) Television & New Media (2018). On data sovereignty see Maggie Walter et al. ed, Indigenous Data sovereignty and Policy.

97 Data disaggregation is one of the six principles identified by the UN Office of the High Commissioner for Human Rights (OHCHR) in its guidance note for a human rights-based approach to data collection: see OHCHR, *A Human Rights-Based Approach to Data-Leaving No One Behind in the 2030 Agenda For Sustainable Development* (2018). In healthcare in particular, it is requested by the CESCR as part of the state obligation to protect the right to health: see CESCR, *General Comment N°14, *at 57, requiring disaggregation on the prohibited grounds of discrimination. In addition to ethnicity that is here our focus, the CESCR states for instance that “[t]he disaggregation of health and socio-economic data according to sex is essential for identifying and remedying inequalities in health.” at 20. On the interplay between equity and disaggregation on the basis of race/ethnicity in particular, see Tina J. Kauh, Jen’nan Ghazal Read, A.J. Scheitler, *The Critical Role of Racial/Ethnic Data Disaggregation for Health Equity*, 40 Popul Res Policy Rev**,** 1–7 (2021). https://doi.org/10.1007/s11113-020-09631-6.

98 Regulation (EU) 2016/679 of the European Parliament and of the Council of Apr. 27, 2016 on the protection of natural persons with regard to the processing of personal data and on the free movement of such data, and repealing Directive 95/46/EC (General Data Protection Regulation) (GDPR), art. 9(1) and exceptions at 9(2) (h) and (i).

99 See for instance Eurotransplant. Among the articles related to ML for clinical transplantation and cited by Connor et al, *The Future Role of Machine Learning in Clinical Transplantation,* those using European data do not use race/ethnicity as variable. See also for example Jesper Kers et al., *Deep Learning-based Classification of Kidney Transplantation Pathology: A Retrospective, Multicentre, Proof-of-Concept Study*, 4(1) The Lancet Digital Health E-18 (2021), acknowledging that « [a]ll participating institutions are from western Europe and we did not include data on ethnicity, since collection of this kind of information is illegal in the Netherlands and not routinely performed in Germany. The same holds true in the French context, see for instance Marc Raynaud et al*., Dynamic Prediction of Renal Survival Among Deeply Phenotyped Kidney Transplant Recipients Using Artificial Intelligence: An Observational, International, Multicohort Study*, 3(12) The Lancet Digital Health E795 (2021).

100 Among the cited articles in the above study from Connor et al. for instance, Mark E, Goldsman et al. *Using Machine Learning and an Ensemble of Methods to Predict Kidney Transplant Survival*, 14 PLoS One e0209068 (2019), selected the ‘recipient ethnicity category’ in their proposed model to predict kidney transplant survival. See also Topuz K, et al., *Predicting Graft Survival Among Kidney Transplant Recipients: A Bayesian Decision Support Model*, 106 Decis Support Syst. 97 (2018), using donor and recipient ethnicity categories in their study on predicting graft survival among kidney transplant recipients (see annex 1). Miller PE et al*., Predictive Abilities of Machine Learning Techniques May be Limited by Dataset Characteristics: Insights from the UNOS Database*. J Card Fail*.* 2019;25:479–483. (donor ethnicity).

101 See a. o. Stephanie Pappas, *Unraveling the Human Genome: 6 Molecular Milestones*, Live Science 2013, Megan Gannon, ‘Race Is a Social Construct, Scientists Argue’, Scientific American 5 (2016). On the collection of ethnic and racial data in the US see Margo J. Anderson, The American Census: a Social History (Yale University Press, 1990). For a comprehensive article on the use of variables of “race” and “Ethnicity” in American public health, see Vickie M. Mays et al., *Classification of Race and Ethnicity: Implications for Public Health*, 24 Annu Rev Public Health 83 (2003).

102 Vivian Chou, *How Science and Genetics are Reshaping the Race Debate of the 21^st^ Century*, Science in the News, Harvard University Blog, Apr. 17, 2017, https://sitn.hms.harvard.edu/flash/2017/science-genetics-reshaping-race-debate-21st-century/ (last accessed June 1, 2022).

103 Insa M. Schmidt and Sushrut S. Waikar, *Separate and Unequal: Race-Base Algorithms and Implications for Nephrology*, in 32 JASN (2021) 529, at 529, explaining that these studies were criticized for being based of small sample sized and questionable anthropometric measurements and assuming race as a sufficient proxy.

104 On the use of race in public health to shed light on variations in health, improve the delivery of health services in the US, see V M. Mays et al., *supra*. The ECtHR protects the right of minorities to free self-identification (see ECtHR Molla Sali v. Greece, No 20452/14, at 157 (2018)). Self-identification is also one of the principles defended by the OHCHR in its guidance note on a human rights-based approach to data see *OHCHR, A Human Rights-Based Approach to Data- Leaving no one behind in the 2030 agenda for sustainable development*.

105 See *‘CHEZ Razpredelenie Bulgaria’ AD v Komisia za zashtita ot diskriminatsia,* C-83/14 EU:C:2015 (2015).

106 Gerards and Xenidis, *Algorithmic Discrimination in Europe* at 67–68, explaining the inconsistency of the CJEU’s approach to this form of discrimination.

107 Bruce A. Julian et al., *Effect of Replacing Race With Apolipoprotein L1 Genotype in Calculation of Kidney Donor Risk Index*, 17 American Journal of Transplantation 1540 at 1546 (2017). See also Krista L. Lentine and Roslyn .B. Mannon, *Apoliprotein L1: Role in the Evaluation of Kidney Transplant Donors*, 29(6) Curr Opin Nephrol Hypertens 645 (2020), Barry I. Freedman et al., *APOL1 Genotype and Kidney Transplantation Outcomes from Deceased African-American Donors*, 100 Transplantaiton 194 (2016).

108 Julian et al, *Effect of Replacing Race With Apolipoprotein L1 Genotype in Calculation of Kidney Donor Risk Index*, at 1547.

109 It does not completely explains the increased risk of allograft outcomes: Girish N. Nadkarni et al., *Apol1: A Case in Point for Replacing Race with Genetics*, 91 Kidney International 766 at 769 (2017). On the need to be attentive to structural racism as part of the risks incurred by Black Americans see Jessica P. Cerdeña et al., *APOL1, Black Race, and Kidney Disease: Turning Attention to Structural Racism*, AJKD 2021. See also Insa M Schmidt et al., *Separate and Unequal: Race-Base Algorithms and Implications for Nephrology*, in 32 JASN 529 at 529 (2021).

110 See GDPR, which affords special protection against the processing of special categories of data, including on racial or ethnic origin, genetic data and data concerning health: art. 9 (h) and (i), 3 and 4. On genetic testing and human rights in Europe see the Additional Protocol to the Convention on Human Rights and Biomedicine, concerning Genetic Testing for Health Purposes, Strasbourg, Nov. 27, 2008 ETS 203, entered into force on July 1, 2018, but has very few ratifications so far. Providing that most kidneys come from deceased donors and are most likely allocated through an equitable system, one could ask if the consent to the retrieval of genetic information on a deceased body can be implied from that person’s (explicit) consent to organ donation; or if a proper consent would be needed. Although these issues will affect the availability of organs and therefore, indirectly in a context of scarcity, the equitable access to organs, it does not properly relate to the use of algorithms and falls beyond the scope of this paper.

111 Margaret Berrigan et al., *Opinions of African American Adults About the Use of Apoliprotein L1 (Apol1) Genetic Testing in Living Kidney Donation and Transplantation*, 21 Am J Transplant 1197 (2020).

112 Oviedo convention, art. 11, see also art. 12.

113 On the obligation to collect specific data as part of the right to health see Claire Lougarre, The right to health: Legal content through supranational monitoring (PhD thesis, UCL 2015) at 92–93.

114 European Commission, Proposal for a Regulation of the European Parliament and the Council on the European Health Data Space, COM(2022)197 final, May 3, 2022. Other initiatives include the Data Governance Act (Proposal for a Regulation of the European Parliament and of the Council on European Data Governance, COM/2020/767 final, Nov. 25, 2020) and the Data Act (Proposal for a Regulation of the European Parliament and the Council on harmonized rules on fair access and use of data, COM/2022/68 final, Feb. 23, 2022). These are market-based instruments.

115 Proposal of Artificial Intelligence Act, art. 10. But art. 10 does not refer to discrimination or equity, rather using the more technical notion of bias. Compromise texts do not change this. The first compromise text (Presidency compromise text, Nov. 29, 2021) added to the list of prohibited practices the placing on the market, putting into service or use of AI system that exploits any of the vulnerabilities… due to ‘social or economic situation…’ (art. 5-1(b)).

116 For high-risk AI systems, Proposal of Artificial Intelligence Act, art. 10 (2). See also Convention 108+, inspiring from national legislations on data protection to impose the quality of data (art. 5).

117 Proposal of Artificial Intelligence Act, art. 10(5).

118
*Id*. 10 (4).

119
*Id*. at 3. See also art. 3(44)(c) of the Compromise text, adding ‘breach of obligations under Union law intended to protect fundamental rights’ in the definition of ‘serious incidents’, see also art 9(2)(a). The text will likely evolve but it will be interesting to follow up on the broader consideration to human rights in the definitive text.

120 Barocas and Selbst, *Big Data’s Disparate Impact*, at 104.

121 As Michael Vaele and Reuben Binns, *Fairer Machine Learning in the Real World: Mitigating Discrimination Without Collecting Sensitive Data*, Big Data & Society 1 (2017), at 2–3 frame it, “[m]odel choice itself can be political.”

122 See section II. In order not to exceed their margin of appreciation, countries need to manage a fair balance of interests in the wording of the ECtHR. In international human rights law, countries must take reasonable steps to achieve the resource allocation dilemma (which goes beyond priority setting). This test of reasonableness remains subject of debate. For a doctrinal discussion on this test in the context of the right to health see John Tobin, The Right to Health in International law (OUP 2012) at 237.

123 In their referential paper on algorithmic biases, going beyond the data challenge, Barocas and Selbst (*Big Data’s Disparate Impact*, at 678) have differentiated between target and class labels: ‘Target variable defines what data miners are looking for’, while ‘class labels’ are ‘all possible values of the target variable into mutually exclusive categories’.

124 See Additional Protocol concerning Transplantation, art. 17. And providing that the body is treated with respect (art. 18).

125 Non-directed living donations being rare in Europe, see data from Newsletter Transplant International, European Directorate for the Quality of Medicine Healthcare (EDQM).

126 Eurotransplant Manual, Chapter 4— –Kidney (ETKAS and ESP), version 3 Nov. 3,ember 2020.

127 An HLA bonus, on top of a pediatric bonus (Scandiatransplant also prioritizes pediatric patients when a suitable kidney is available and the donor is less than 40 years old). Such policy can be considered as a “vulnerability” approach in the sense that human rights law recognizes children as vulnerable persons in general, which requires authorities to pay particular attention to their situation. From an ethical perspective on resource allocation, see Robert M Veatch, Transplantation Ethics (Georgetown University Press 2000), on the justice-over-a-lifetime argument, and Keren Ladin and Douglas W. Hanto, *Rational Rationing or Discrimination: Balancing Equity and Efficiency Considerations in Kidney Allocation* 11(11) Am J Transplant 2317 (2011) on the fair-innings argument. See also Alan Williams, *Intergenerational Equity: An Exploration of the “Fair Innings” Argument*, 6 J. Health Econ. 117 (1997). For a critical perspectives on these arguments supporting pediatric priority see Laura Capitaine et al., *Pediatric Priority in Kidney Allocation: Challenging its Acceptability*, 27 Transplant International 533 (2014).

128 Eurotransplant Manual Chapter 4—Kidney (ETKAS and ESP), version Nov. 3 2020.

129 As explained in Mary Anne Bobinski, David Orentlicher, I. Glenn Cohen, Mark A. Hall l, Bioethics and public health law 432-449 (4th edn, 2018). On the luck egalitarianism theory of distributive justice applied to organ allocation, see also Andreas Albertsen, *Priority to Organ Donors: Personal Responsibility, Equal Access and the Priority Rule in Organ Procurement*, 51 Diametros 137 (2017).

130 Corrinne Purtill, *How AI Changed Organ Donation in the US*, Quartz, Sept. 10, 2018.

131 Eurotransplant tries to mitigate this competition by the awarding of national balance points for kidney exchanges: Eurotransplant Manual Chapter 4.10.4. at 28–29.

132 Stefanos A. Zenios et al., *Evidence-Based Organ Allocation*, 107 The American Journal of Medicine 52 (1999).

133 Keren Ladin and Douglas Wayne Hanto, *Rational Rationing or Discrimination: Balancing Equity and Efficiency Considerations in Kidney Allocation,* 11 American Journal of Transplantation 2317, at 2317 (2011).

134 There is however a statute prohibiting discrimination on this basis, *see* Benjamin Eidelson, *Kidney Allocation and the Limits of the Age Discrimination Act*, 122(6) Yale Law Journal 1635, at 1636 (2013).

135 EU Charter of Fundamental Rights, art. 20.

136
*Id*. art. 21. See also art. 25 on the rights of the elderly and art. 26 on the integration of persons with disabilities.

137 Recommendation CM/Rec(2014)2 of the Committee of Ministers to member States on the promotion of human rights of older persons (2014), points 6 and 7.

138 Committee on Bioethics (DH-BIO), Strategic Action Plan on Human Rights and Technologies in Biomedicine.

139
*Id*.

140 See for example Govind Persad, *Evaluating the Legality of Age-based Criteria in Health Care: From Non-discrimination and Discretion to Distributive Justice*, 60 Boston College Law Review 889 (2019), defending a lifetime justice approach.

141 Capitaine et al., *Pediatric priority in kidney allocation*.

142 Titti Mattson, *Editorial: Digitalisation and Artificial Intelligence in European Healthcare*, 26(4) EJHL 285 at 286 (2019).

143 See for instance ECtHR, Molla Sali v. Greece, at 133.

144
Delphine Tharaud, Droit de la non-discrimination 136 (2021).

145 See Biao v. Denmark*,* at 103.

146 Eurotransplant Senior Program (ESP). For a recent evaluation of the program see Thomas Schachtner et al., *Two Decades of the Eurotransplant Senior Program: The Gender Gap in Mortality Impacts Patient Survival After Kidney Transplantation*, 13(6) Clinical Kidney Journal 1091 (2020).

147 National Down Syndrome Society (NDSS), Need for Legal Protection Against Organ Transplant Discrimination on the Basis of Disability available at [https://www.ndss.org/wp-content/uploads/2017/10/MD-SB792-SampleFactSheet.pdf] “Although the Americans with Disabilities Act outlaws discrimination on the basis of disability, including discrimination by doctors, many health providers fail to recognize that this can apply to discriminatory denials of organ transplants.”

148 Charlotte Woodward Organ Transplant Discrimination Prevention Act (H.R.1235/S.3301), in order to reinforce and make effective the Americans with Disability Act of 1990. For more, see National Council on Disability*,*  *Organ Transplant Discrimination Against People with Disabilities*, Sept. 25, 2019, explaining that “in some cases, organ transplant centers may categorically refuse to evaluate a patient with a disability as a candidate for transplant.” This is partly due to the assumption that patients with mental health disabilities will be unable to comply with necessary postoperative care.

149 In France for example, some have denounced the principle of local attribution because of the disparities in access. In a region like Paris and the surroundings, demography is a factor to consider (younger people with less risks of dying from cardio-vascular issues and therefore less people in brain death. Providing that most organs are recovered from deceased donors in brain death, patients in need for transplant in this region would have less chances to get a transplant in a certain time than in other regions). The disadvantage is both on access and quality of the transplant. Those who are well informed and can afford it will register in other regions, but the less privileged cannot afford it, see Marielle Ammouche, *Inégalités d’accès à la greffe: une association dénonce le principe du rein local* [https://www.egora.fr/actus-medicales/chirurgie/40037-inegalites-d-acces-a-la-greffe-une-association-denonce-le-principe] (last accessed June 1, 2022). See also Eurotransplant regional data on waiting lists and transplants.

150 Bart Smeulders et al., *Data and Optimisation Requirements for Kidney Exchange Programs*, 27(2) Health Informatics Journal 1 at 3 (2021).

151 Marry De Klerk et al., *The Dutch National Living Donor Kidney Exchange Program*, 5(9) American Journal of Transplantation 2302 (2005). More generally, see program developed by Alvin E. Roth et al., *Kidney Exchange*, 119(2) Quarterly Journal of Economics 457 (2004) and their subsequent work.

152 Countries such as Spain, the Netherlands, the United Kingdom or Sweden have effectively developed kidney exchange programs. France’s 2021 bioethical legislation, which increased the maximum amount of pairs to six pairs instead of two pairs since 2011 will facilitate matching and make the program effective. Kidney exchange programs are not, however, as developed as in North-America. For data on transplantation activity, *see* the annual Newsletters Transplant International. On the development of kidney exchange programmes in Europe, see Péter Biró et al., *Building Kidney Exchange Programmes in Europe—An Overview of Exchange Practice and Activities*, 103 Transplantation 1514 (2019). For cross-border exchange programs, *see* also the Council of Europe Resolution CM/Res(2015)11 on establishing harmonized national living donor registries with a view to facilitating international data sharing.

153 ENCKEP, Modeling and Optimization in European Kidney Exchange Programmes. Second Handbook of Working Group 1 in the COST Action CA15210: European Network for Collaboration on Kidney Exchange Programmes (ENCKEP), 24 Jan. 2019, at 13. Available at [https://www.enckep-cost.eu/news/news-modelling-and-optimisation-in-european-kidney-exchange-programmes-156]. In 2018, a first international cross-kidney transplantation was undertaken in the context of the SAT (between Italy and Spain) and following international crossover protocol: see EKHA (European Kidney Health Alliance), *Italy and Spain Achieved First International Cross-Kidney Transplantation*, Sept. 5 2018.

154 For a discussion on data requirements for Kidney exchange programs in Europe *see* Bart Smeulders et al., *Data and optimisation requirements for Kidney Exchange Programs*.

155 Except if a legislation were to authorize deceased donors to name particular recipients of their organs after death, *see* Uniform Anatomical Gift Act (2006) in the United States.

156
*See for example data from Newsletter Transplant International: European Directorate for the Quality of Medicine Healthcare (EDQM) (2021).*

157 The Oviedo Convention and its protocol concerning transplantation do not make distinctions regarding the protection of living donors depending on the direct donation to an identified recipient or the participation in a kidney exchange program with multiple pairs. In consequence, the specificity of the living donor’s consent (…) is only preserved if the recipient she designated but is incompatible with, receives a transplantation in return (unless unforeseen event). That is why the removals would need to happen simultaneously (which is a logistic constraint playing on the algorithm—Most European countries require simultaneous surgeries: see Péter Biró et al.,* Building Kidney Exchange Programmes in Europe* at 1518) unless organs from altruistic or deceased donors were inserted into the chains (but it raises other issues). However, it belongs to the authorities to allocate equitably the organs inside the pools, including to decide between different acceptable combinations.

158 Rachel Freedam et al., *Adapting a Kidney Exchange Algorithm to Align with Human Values*, AIES '18: Proceedings of the 2018 AAAI/ACM Conference on AI, Ethics, and Society, Dec. 2018.

159 See the very instructive report ENCKEP, Modelling and Optimisation in European Kidney Exchange Programmes, at 13.

160 Rachel Freedam et al., *Adapting a Kidney Exchange Algorithm to Align with Human Values*, AIES '18: Proceedings of the 2018 AAAI/ACM Conference on AI, Ethics, and Society, Dec. 2018.

161
*Id.*

162 Cultural appropriateness is part of the requirement of Acceptability of implementing measures of the right to health: see CESCR, *General Comment No. 14,* at 12(c). For a discussion on this topic, see Audrey Lebret, *Cultural Appropriateness of Artificial Intelligence in Health Care* in Social Justice and Artificial Intelligence, Karine Gentelet (PUL 2023), forthcoming.

163 Oviedo convention, art. 5, Additional Protocol on Transplantation, art. 13.

164 Rajkomar et al.,* Ensuring Fairness in Machine Learning to Advance Health Equity* identifying four categories of bias in healthcare, bias in model design, in training data, in interactions with clinicians and in interactions with patients.

165 US Studies have shown knowledge deficiencies on disparities in access to kidney transplant among nephrologists and dialysis staff. For a review and references Park et al., *A scoping review of inequities in access to organ transplant in the United States.*

166 Alvin Roth, Kidney exchange 2000–2015: *Algorithms and Incentives*, Simons Institute for Theoretical Computer Science, Berkeley, Nov., 16 2015, available at: [https://simons.berkeley.edu/talks/alvin-roth-2015-11-16] (last accessed May 25, 2022).

167 For the countries allowing organ recovery after controlled circulatory death. See Mar lomero et al., *Donation After Circulatory Death Today: An Updated Overview of the European Landscape*, 33 Transplant International 76 (2020).

168 See for instance Katherine Ross et al., *Sociodemographic Determinants of Waitlist and Posttransplant Survival Among End-Stage Liver Disease Patients*, 17 American Journal of Transplantation 2879 (2017), noting that proposed changes in liver organ distribution policy in the US in order to minimize the differences in access between regions exposed a paradox as regions having ‘excess’ organs also had much higher waitlist mortality rates. The insensitiveness to sociodemographic disparities could, in the authors’ view, ‘significantly worsen transplant access for already disadvantaged populations’. See also Keren Ladin, Gregory Zhang and Douglas W Hanto, *Geographic Disparities in Liver Availability: Accidents of Geography, or Consequences of Poor Social Policy?*, in 17 American Journal of Transplantation 2277 (2017), noting ‘Variation in liver availability may not be an “accident of geography” but rather a byproduct of disadvantage’.

169 See for example les états généraux de la bioéthique in France, broad consultation of the population before the revision of biolaws. This would require prior pedagogy on algorithmic development.

170 See for example Héloïse Cardinal and collaborators’ project,* The development of a clinical decision support tool integrating machine learning approaches...* .

171 Connor et al., *The Future Role of Machine Learning in Clinical Transplantation *at 725.

172
*Id*. at 732.

173 Philip Hacker, *Teaching Fairness to Artificial Intelligence: Existing and Novel Strategies Against Algorithmic Discrimination Under EU Law*, 55 Common Market Law Review 1143 (208).

174 See Sections II and III.A.2.

175 Barocas and Selbst, *Big Data’s Disparate Impact*, at 691.

176 Cynthia Dwork et al., *Fairness Through Awareness,* 3 Proc. Innovations Theoretical Computer SCI. Conf. 214 app. at 226 (2012) ("Catalog of Evils"), Barocas and Selbst, *Big Data’s Disparate Impact*, at 692.

177 See Indrė Žliobaitė and Bart Custers, *Using Sensitive Personal Data May be Necessary for Avoiding Discrimination in Data-Driven Decision Models*, 24 Artificial Intelligence and Law 183. Besides, the removing of problematic correlations with sensitive categories may affect the accuracy of the model: see Toon Calders and Sicco Verwer, *Three Naive Bayes Approaches for Discrimination-free Classification*, 21 Data Mining and Knowledge Discovery 277 (2010), see also Vaele and Binns, *supra*, at 4.

178 Section III.B.1.a.

179 Vaele and Binns, *Fairer machine learning in the real world...,* at 3–4 mention several tests, although they usually cannot be taken at the same time.

180
*Id*. On data protection see also Council of Europe, Convention for the Protection of Individuals with regard to Automatic Processing of Personal Data (ETS No. 108) ‘Convention 108+’ (1981, as amended by Protocol 223 in 2018) and its additional Protocols.

181 Hacker, *Teaching fairness to artificial intelligence...* at 1150.

182
*Id*., see also James Grimmelmann and Daniel Westreich, *Incomprehensible Discrimination*, 7 California Law Review Online 164 (2017), at 168.

183 Judgment *Jyske Finans A/S v Ligebehandlingsnavnet, Acting on Behalf of Ismar Huskic* C-668/15 EU:C:2017:278 (2017). See Gerards and Xenidis, *Algorithmic Discrimination in Europe*, at 71.

184 Gerards and Xenidis, *Algorithmic Discrimination in Europe*.

185 Gerald Koon, *Premie autoverzekering op basis regio en postcode [Premiums for Car Insurance Based on Region and Postal Code],* CONSUMENTENBOND [DUTCH CONSUMER ORGANISATION] (Nov. 29, 2017), cited by Janneke Gerards and Frederik Zuiderveen Borgesius, *Protected Grounds and the System of Non-discrimination Law in the Context of Algorithmic Decision-Making and Artificial Intelligence*, 20(1) Colo. Tech. L.J. (2022), at 3.

186 Sandra Wachter, *The Theory of Artificial Immutability: Protecting Algorithmic Grounds Under Anti-Discrimination Law*, 97 Tul. L. Review. XX (2022-2023) forthcoming, Available at SSRN: https://ssrn.com/abstract=4099100 or http://dx.doi.org/10.2139/ssrn.4099100, at 5-6.

187
*Id*. makes this distinction at 7.

188 Matthias Leese, *The New Profiling: Algorithms, Black Boxes, and the Failure of Anti-Discriminatory Safeguards in the European Union*, 45 Security Dialogue 501 (2014), at 503 talks about ephemeral groups.

189 Wachter, *The Theory of Artificial Immutability...*, at 13.

190 See section II.

191 When it comes to the development of Protocol 12 to the ECHR, the Assembly, arguing for the inclusion of sexual orientation, noted that ‘[the grounds included] have been selected because we have learnt to regard discrimination on these grounds to be the most insidious and obnoxious forms of discrimination’: Draft Protocol No.12 to the European Convention on Human rights (Doc 8614), Jan. 14, 2000, at 35. It appeared from the preparatory work that the inclusion of new grounds was found unnecessary or even potentially counter-productive because it could give rise to ‘unwarranted a contrario interpretations as regards discrimination based on grounds not so excluded” (at 18).

192 This observation also applies for the consideration of multiple and intersectional forms of discrimination previously discussed.

193 Vaele and Binns, *Fairer machine learning in the real world...* at 3-4.

194 See Hoogendijk v. the Netherlands In this respect, the amicus curiae procedure has largely contributed to providing the judges with the necessary contextual elements for the decision. Nevertheless, as seen above, the realization of statistic studies is regulated in Europe both in terms of the right to protection of personal data and the collection of ethnic data. This is why the preventive approach is preferable.

195 A few scholars have engaged in the discussion. See in particular Sandra Wachter, who develops a theory of artificial immutability in her forthcoming article *The Theory of Artificial Immutability: Protecting Algorithmic Grounds Under Anti-Discrimination Law*. *See* also Gerards and Zuiderveen Borgesius, *Protected grounds and the system of non-discrimination law in the context of algorithmic decision-making and artificial intelligence*.

196 GDPR, art. 22(1): “The data subject shall have the right not to be subject to a decision based solely on automated processing, including profiling, which produces legal effects concerning him or her or similarly significantly affects him or her.”

197 See a.o. Hacker, *Teaching fairness to artificial intelligence...*, Frederik Zuiderveen Borgesius, *Discrimination, Artificial Intelligence and Algorithmic Decision Marking,* study for the Council of Europe (2018). Peoples’ digital self-determination is also an interesting framework.

198 Besides, the issue of articulation of the multiple regulations at the EU level such as the-Proposals of AI Act, of Health Data Space, GDPR etc- still needs to be addressed.

199 Council of Europe, News 2022, Congress contributes to the Council of Europe’s draft Convention on Artificial Intelligence, Strasbourg, Sept. 22, 2022, available at: https://www.coe.int/en/web/congress/-/congress-contributes-to-the-council-of-europe-s-draft-convention-on-artificial-intelligence (last accessed Sept. 24 2022)

200 See Lorna McGregor et al., *International Human Rights Law as a Framework for Algorithmic Accountability*, 68(2) International & Comparative Law Quarterly 309 (2019).

201 Proposal of Artificial Intelligence Act, art. 6 (1)(a). On the definition of medical devices see Regulation (EU) 2017/745 of the European Parliament and of the Council of 5 April 2017 on medical devices, amending Directive 2001/83/EC, Regulation (EC) No 178/2002 and Regulation (EC) No 1223/2009 and repealing Council Directives 90/385/EEC and 93/42/EEC. See Compromise text, §§30-31, but see that the compromise text excluded “software” from the definition of the definition of “Artificial Intelligence” of the original proposal, art. 3 (1).

202 See generally Council of Europe, Algorithms, and AI developement (last accessed June 1, 2022).

203 On the notion of structural obligation in international human rights law, see Anna Glazewski, Les ‘obligations structurelles’ de l’Etat au regard du droit international des droits de l’homme- Recherche sur une nouvelle catégorie juridique (PhD thesis, Panthéon-Assas University 2018).

204 CESCR, *General Comment No. 14, *at 20.

^205^
See for example Ladin et al., *Geographic, Disparities in Liver Availability*… showing that redistribution may not be the most relevant option—and could even exacerbate existing inequalities—when disparities in organ allocation are linked to risks of violent death.

206 Audrey Lebret, *A Global Human-Rights Approach to Medical Artificial Intelligence* in AI in eHealth: Human Autonomy, Data Governance and Privacy in Healthcare, Marcelo Corrales Compagnucci et al. (CUP 2022).

